# Improving the accuracy of the FMO binding affinity prediction of ligand-receptor complexes containing metals

**DOI:** 10.1007/s10822-023-00532-2

**Published:** 2023-09-25

**Authors:** R. Paciotti, A. Marrone, C. Coletti, N. Re

**Affiliations:** Department of Pharmacy, Università “G. D’Annunzio” Di Chieti-Pescara, Chieti, Italy

**Keywords:** FMO2, FMO3, PIE, Pair interaction energy, Binding energy, Screened charges, DNA G-quadruplex, Biscarbene-Au(I) ligands, Metal-based drugs

## Abstract

Polarization and charge transfer strongly characterize the ligand-receptor interaction when metal atoms are present, as for the Au(I)-biscarbene/DNA G-quadruplex complexes. In a previous work (*J Comput Aided Mol Des*2022, 36, 851–866) we used the ab initio FMO2 method at the RI-MP2/6-31G* level of theory with the PCM [1] solvation approach to calculate the binding energy (*ΔE*^*FMO*^) of two Au(I)-biscarbene derivatives, [Au(9-methylcaffein-8-ylidene)_2_]^+^ and [Au(1,3-dimethylbenzimidazole-2-ylidene)_2_]^+^, able to interact with DNA G-quadruplex motif. We found that *ΔE*^*FMO*^ and ligand-receptor pair interaction energies (*E*^*INT*^) show very large negative values making the direct comparison with experimental data difficult and related this issue to the overestimation of the embedded charge transfer energy between fragments containing metal atoms. In this work, to improve the accuracy of the FMO method for predicting the binding affinity of metal-based ligands interacting with DNA G-quadruplex (Gq), we assess the effect of the following computational features: *(i)* the electron correlation, considering the Hartree–Fock (HF) and a post-HF method, namely RI-MP2; *(ii)* the two (FMO2) and three-body (FMO3) approaches; *(iii)* the basis set size (polarization functions and double-ζ vs. triple-ζ) and *(iv)* the embedding electrostatic potential (ESP). Moreover, the partial screening method was systematically adopted to simulate the solvent screening effect for each calculation. We found that the use of the ESP computed using the screened point charges for all atoms (ESP-SPTC) has a critical impact on the accuracy of both *ΔE*^*FMO*^ and *E*^*INT*^, eliminating the overestimation of charge transfer energy and leading to energy values with magnitude comparable with typical experimental binding energies. With this computational approach, E^INT^ values describe the binding efficiency of metal-based binders to DNA Gq more accurately than *ΔE*^*FMO*^. Therefore, to study the binding process of metal containing systems with the FMO method, the adoption of partial screening solvent method combined with ESP-SPCT should be considered. This computational protocol is suggested for FMO calculations on biological systems containing metals, especially when the adoption of the default ESP treatment leads to questionable results.

**Supplementary information:**

The online version contains supplementary material available at 10.1007/s10822-023-00532-2.

## Introduction

The interaction between biological systems and small molecules containing metal atoms is difficult to describe with standard molecular mechanics (MM) methods, widely used in computer assisted drug discovery (CADD). In these environments, polarization, charge-transfer and dispersion interactions are broadly present and only quantum mechanics (QM) methods can describe these phenomena and their contribution to the binding energy with enough accuracy.

One of the most interesting QM approaches to study large biological molecules is the fragment molecular orbital (FMO) method that allows the splitting of the target system in many atom groups (e.g., one amino acid), named fragments, and the computation of the relative interaction [1].

The FMO2 is the most used approach where the total energy is basically computed as the sum of the energy of each fragment and the interaction energy between each pair of fragments [2]. The latter term, named pair interaction energy (PIE), can be decomposed by means of energy decomposition analysis (EDA) into electrostatic energy (*E*^*es*^), exchange energy (*E*^*ex*^), charge transfer energy (*E*^*ct*^), dispersion energy (*E*^*dis*^) e solvation energy (E^solv^) [3, 4]. When one fragment is a ligand, the sum of PIEs with respect to all other fragments, hereafter named E^INT^, provides an estimation of the ligand binding strength. In this case, PIEDA provides an important insight on the nature of ligand-receptor interactions and a useful descriptor in quantitative structure-activity relationship studies [5].

**Fig. 1:**
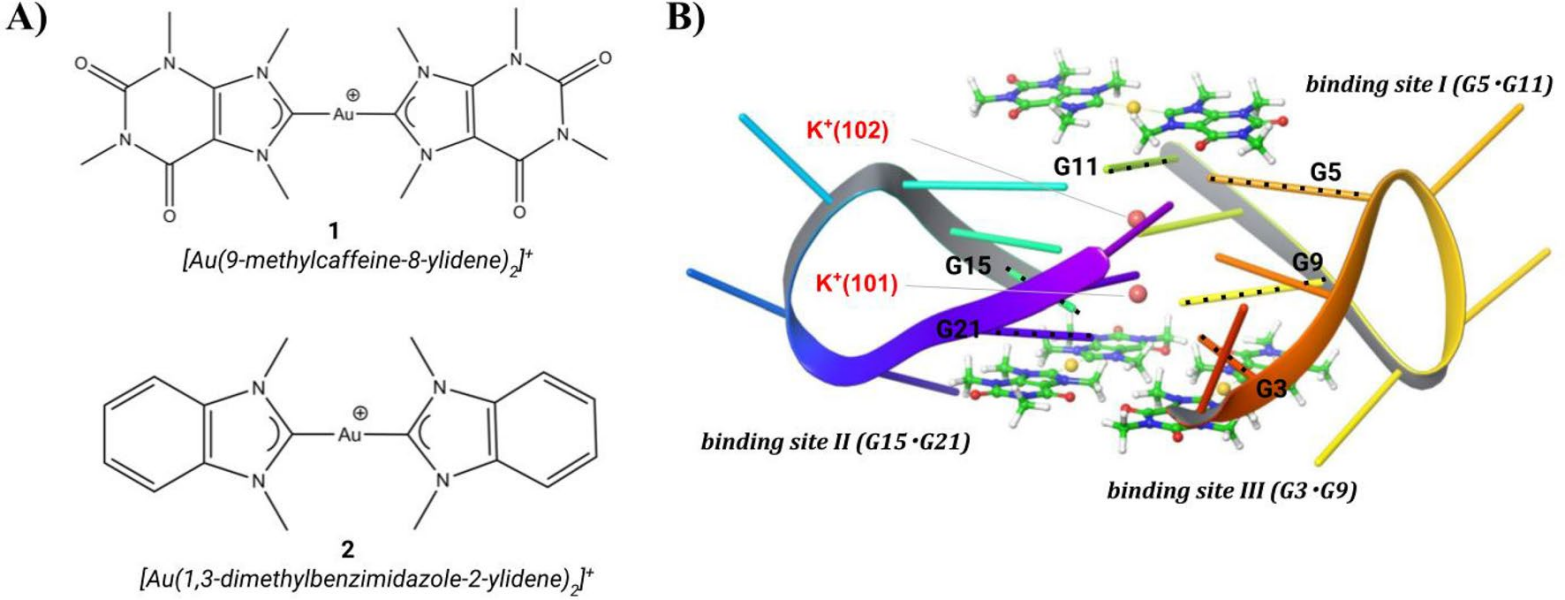
**a** 2D structures of 1, [Au(9-methylcafein-8-ylidene)_2_]^+^, and 2, [Au(1,3-dimethylbenzimidazole-2-ylidene)_2_]. B) Optimized structure of the complex formed by Gq and three molecules of ligand 1 (PDB ID: 5CCW). The guanine pairs G5·G11, G15·G21 and G3·G19 forming the binding sites I, II and III, respectively, are also shown. The K^+^ ions are represented by pink spheres, and ligand 1 molecules are shown in ball and stick style

The FMO2 approach with the implicit solvation method has been widely applied to study biological systems as protein-protein interactions [6, 7], protein-DNA interactions [8], protein structures and stability [9], ligand-receptor interactions [10] and small metal complexes [11]. Systems that can profitably be investigated by the application of the ab initio FMO method are receptors, typically bio-macromolecules, that natively bind metals and/or interact with metallic compounds. One interesting system of this class is represented by the G-quadruplex structure (Gq), a peculiar DNA motif where clusters of four guanines interact via hydrogen bonds and are stabilized by the presence of two K^+^ ions [12, 13] coordinated by the O6 atoms of guanine rings. K^+^ ions also induce polarization and a charge transfer from Gq to the metal ions; such effects can only be correctly evaluated through QM levels of theory. DNA Gq structures were found in several eukaryotic promoters [14] and oncogenes [15], so that specific binders of this DNA motif might act as antitumor agents [16, 17]. Metal complexes, as Au(I) complexes, have been reported as promising candidates to hit the DNA Gq structures [18, 19]. Au(I)-binder/Gq complexes represent typical systems of interest for the application of the FMO method. In a recent work [20], we employed the FMO2 approach to estimate the binding energy (*ΔE*^*FMO2*^) of the complexes formed by DNA Gq and two different biscarbene-Au(I) binders, [Au(9-methylcaffein-8-ylidene)_2_]^+^ and [Au(1,3-dimethylbenzimidazole-2-ylidene)_2_]^+^, hereafter named 1 and 2, respectively (Fig. 1a) [21, 22]. Their affinities for DNA Gq have been measured by performing FRET-melting assay experiments which indicated that ligand **1** is a stronger Gq-binder than 2 [21]. As shown in Fig. 1b, the Au(I)-binders were found to be hosted by Gq in three distinct sites (I, II, and III), according to X-Ray structure obtained for ligand 1 (PDB ID: 5CCW) [23].

On this basis, we applied the procedure reported by Fedorov et al. [24], who consider the ligand-receptor complex and the minimized isolated forms of both ligand and receptor, to estimate and analyze the *ΔE*^*FMO2*^ of ligand 1 and 2 at the three binding sites of DNA-Gq (Fig. 1b). Within this scheme, the destabilization polarization and desolvation energies are considered leading to a more accurate estimation of the binding affinity compared with *E*^*INT*^.

We found that at RI-MP2/6-31G* level of theory (often employed in FMO studies) *ΔE*^*FMO2*^ and *E*^*INT*^ can profitably be used for the ranking of the two Au(I)-binders, even if they assume very large negative values forbidding a quantitative comparison with experimental binding data. We showed that this is to be ascribed to the large PIE values for the K^+^ containing fragments, caused by an overestimation of the explicit embedded charge transfer (CT) energy, *Tr(ΔD*^ij^*V^*ij*^*)*, in the presence of the metal-based ligand [20].

Typically, in FMO calculations, depending on the distance between two fragments, the embedding electrostatic potential (ESP), *V*^*ij*^, is computed by using Mulliken point charges (ESP-PTC approximation) or by considering the two-electron integral contributions to the ESP (ESP2) [25].

Thus, the way of calculating the ESP within the FMO method might significantly affect the magnitude of the explicit embedded CT energy. To this aim, the adoption of ESP-PTC computed for all atoms using the screened point charges (ESP-SPTC) might represent a promising option [26].

A further way to reduce the overestimation of the CT energy is to adopt the three-body approach, FMO3 [27]. It has been reported that the three-body interactions implemented in FMO3/EDA can correct the overestimation of the CT energy affecting the FMO2/PIEDA approach [28]. Finally, the basis set type, e.g., double-ζ or triple-ζ, and the presence of polarization functions can significantly affect the accuracy of the electronic structure picture that might reflect in an improved estimate of CT energy.

In this work, the accuracy of the FMO method for predicting the binding affinity of metal-based ligands interacting with DNA Gq was improved by refining the description of the CT energy. To this purpose, we evaluated the impact of the following computational features on the FMO results: *(i)* the effect of electron correlation, considering the Hartree-Fock (HF) and a post-HF method as RI-MP2; *(ii)* the effect of the two- (FMO2) and three-body (FMO3) approaches; *(iii)* the impact of the basis set type (double-ζ vs. triple-ζ and the effect of polarization functions) and *(iv)* the effect of the ESP-SPCT. The partial screening method to simulate the solvent screening effect was adopted for all calculations.

Several FMO computational schemes were analysed and compared with previous work or available experimental data leading to the identification of the best computational settings to study the Au(I)-biscarbene/Gq complexes. The tuning of the FMO method suggested in this study is expected to be extendable to other biological systems including metal atoms.

## Method

### Theoretical background

The FMO2 energy of a molecular system composed by N fragments, using an implicit solvation method, is computed by following equation:

1 $${E}^{FMO2}= \sum _{i=1}^{N}{E}^{{\prime }}+\sum _{i>j}^{N}{E}_{ij}^{PIE}$$

where E′ can be divided into the internal solute energy of the fragment, E″, and its solvation energy, ${E}_{i}^{SOL}$, as

2 $${E}^{{\prime }}= {E}^{{\prime }{\prime }}+ {E}_{i}^{SOL}$$

${E}_{ij}^{PIE}$ (Eq. 1) is computed as follows from the difference between the internal solute energy of the *ij* pair and those of the single fragments *i* and *j*:

3 $$E_{{ij}}^{{PIE}} = \left( {E_{{ij}}^{{''}} - E_{i}^{{''}} - E_{j}^{{''}} } \right) + Tr\left( {\Delta D^{{ij}} *V^{{ij}} } \right) + E_{{ij}}^{{sol}}$$

where ${E}_{ij}^{sol}$ is the solvation energy of the *ij* pair with respect to those of the monomers *i* and *j*, ∆D^*ij*^ is the density matrix difference of the dimer *ij* and the sum of monomers *i* and *j* electron densities and V^ij^ is the matrix of the contribution of all the other fragments to the electrostatic potential acting upon the dimer *ij* [24].

The total energy can be written as

4 $${E}^{FMO2}= \sum _{i=1}^{N}{E}^{{\prime }}+\sum _{i>j}^{N}\left({E}_{ij}^{{\prime }{\prime }}-{E}_{i}^{{\prime }{\prime }}-{E}_{j}^{{\prime }{\prime }}\right)+\sum _{i>j}^{N}Tr\left({\Delta }{D}^{ij}*{V}^{ij}\right)+\sum _{i>j}^{N}{E}_{ij}^{sol}$$

The first two terms of Eq. 4 are also known as internal energies, *E*^*internal*^, and the third one as the embedding energy, *E*^*emb*^ [29]:

5 $${E}^{internal}= \sum _{i=1}^{N}{E}^{{\prime }}+\sum _{i>j}^{N}\left({E}_{ij}^{{\prime }{\prime }}-{E}_{i}^{{\prime }{\prime }}-{E}_{j}^{{\prime }{\prime }}\right)$$

6 $${E}^{emb}= \sum _{i>j}^{N}Tr\left({\Delta }{D}^{ij}*{V}^{ij}\right)$$

Thus, the total FMO energy, *E*^*FMO2*^, can be written as a function of *E*^*internal*^ and *E*^*emb*^ :

7 $${E}^{FMO2}={E}^{internal}+{E}^{emb}+\sum _{i>j}^{N}{E}_{ij}^{sol}$$

*E*^*internal*^ describes the many-body polarization effect on the two-body interactions, while *Tr(ΔD*^ij^*V^*ij*^*)* is the energy related to inter-fragment charge (electron density) transfer, *ΔD*^*ij*^, under the influence of the embedding potential, *V*^*ij*^, for fragments polarized by ESP. In other words, it quantifies whether surrounding charge distributions of fragments other than *i* and *j* (in ESP, *V*^*ij*^) promote or demote the charge transfer between *i* and *j* in dimer *ij* [29]. Therefore, one expects *E*^*emb*^ to be more sensitive to ESP than *E*^*internal*^ and that the strategy to compute the ESP can significantly affect the magnitude of the CT energy.

Generally, the ESP-PTC approximation is used when the distance between two fragments exceeds the criterion defined by RESPPC keyword (e.g., RESPPC = 2.0 is the default value for FMO2 approach) while the most computational demanding ESP2 is used otherwise [25].

The ESP-PTC can also be computed using the screened point charges for all atoms (ESP-SPTC) adopting the following gaussian dumping function [26]:

8 $$f\left(R\right)=1-a*\text{exp}\left(-b{R}^{2}\right)$$

where *R* is distance between point charges and *a* and *b* are two constant parameters.

PIE can be decomposed in several terms according to PIEDA [3, 4] as

9 $${E}_{ij}^{PIE}={E}_{ij}^{es}+{E}_{ij}^{ex}+{E}_{ij}^{ct+mix}+{E}_{ij}^{disp}+{E}_{ij}^{sol}$$

Applying the procedure presented by Fedorov et al. [24], the FMO2 binding energy, *ΔE*^*FMO2*^, can be computed as

10 $$\triangle E^{{FMO2}} = E_{{LR}}^{{FMO2}} - E_{{R}}^{{FMO2}} - E_{{L}}$$

where *E*_*LR*_, *E*_*L*_ and *E*_*R*_ are the total FMO2 energy of LR and of the isolated ligand and receptor, respectively.

The total energy with the FMO3 approach is defined by the following equation:

11 $$E^{{FMO3}} = \sum\limits_{{i = 1}}^{N} {E^{\prime } } + \sum\limits_{{i > j}}^{N} {E_{{ij}}^{{PIE}} } + \sum\limits_{{i > j > k}}^{N} {\omega _{{ijk}} } E_{{ijk}}^{{PIE}}$$

where the third term represents the three-body (FMO3) corrections to PIE averaged by adopting a normalized weight factor (ω_ij,k_ + ω_kj,i_ + ω_ik,j_ = 1) and the simplest situation is obtained when ω_ij,k_ = ω_kj,i_ = ω_ik,j_ = 1/3 [28].

The total FMO3 PIE can be written as

12 $$\sum\limits_{{i > j}}^{N} {E_{{ij}}^{{PIE*}} } = \sum\limits_{{i > j}}^{N} {E_{{ij}}^{{PIE}} } + \sum\limits_{{i > j > k}}^{N} {\omega _{{ijk}} E_{{ijk}}^{{PIE}} }$$

which, introduced in Eq. (11), gives:

13 $$E^{{FMO3}} = \sum\limits_{{i = 1}}^{N} {E^{\prime } } + \sum\limits_{{i > j}}^{N} {E_{{ij}}^{{PIE*}} }$$

Notably, the *E′* terms (and therefore *E″* and *E*^*SOL*^ terms, Eq. 2) are the same ones introduced in Eq. 1 for the total FMO2 energy. An accurate description of the FMO3 method and of the corresponding equations has been recently reported [28]. E^FMO3^ can now be used to define the FMO3 binding energy, *∆E*^*FMO3*^, according to Eq. 10.

Considering a ligand-receptor complex, LR, where the receptor (R) is composed by N fragments and L is the ligand, considered as an additional fragment, the total PIE of the ligand with the receptor, *E*^*INT*^, is:

14 $${E}_{Li}^{INT}= \sum _{i=1}^{N}{E}_{Li}^{PIE}$$

that can be written as a function of *E*^*emb*^ by combining it with Eq. 6:

15 $${E}_{Li}^{INT}= \sum _{i=1}^{N}({E}_{Li}^{{\prime }{\prime }}- {E}_{i}^{{\prime }{\prime }}-{E}_{L}^{{\prime }{\prime }})+\sum _{i=1}^{N}{E}_{Li}^{emb}+\sum _{i=1}^{N}{E}_{Li}^{sol}$$

### Computational details

The geometry of Gq-1 and Gq-2 complexes were retrieved from previous work [20], using the computational procedure briefly described as follows.

The X-ray structure (PDB ID: 5CCW) [23] of DNA G-q in a complex with three molecules of ligand 1 in three different sites (I, II and III) was refined by using the protein preparation tool [30, 31] and Macromodel [31] while the free ligands, 1 and 2, were optimized at B3LYP/6–311+G** level of theory, adopting the LANL2DZ pseudopotential for Au atoms, by using Gaussian suite [32]. Notably, the terminal RPO_3_ group (5′ position) of the sugar-phosphate DNA backbone has been treated as RPO_4_^2−^.

Each binding pose was evaluated separately to obtain three LR complexes, Gq(I)-1, Gq(II)-1 and Gq(III)-1 complexes. The corresponding adducts for ligand 2 (Gq(I)-2, Gq(II)-2 and Gq(III)-2) were built by manually superimposing the structure of 2 with 1.

Then, the free DNA Gq structure and Gq(I)-l, Gq(II)-1, Gq(III)-1, Gq(I)-2, Gq(II)-2 and Gq(III)-2, were minimized using density functional tight-binding (DFTB) theory, adopting the GFN2-xTB method [33] combined with the GB/SA approach to simulate the solvation effect. DFTB calculations were performed by using the in xTB software [34, 35].

In this work, the optimized geometries of Gq-ligand complexes and of the isolated species were used as input for several FMO single point calculations in order to assess the impact of the following aspects on the accuracy of *E*^*INT*^ and *∆E*^*FMO*^: the level of theory (HF and RI-MP2), the basis set (namely, the effect of polarization functions on heavy atoms and of double-ζ or triple-ζ quality sets), the n-body approach (FMO2 and FMO3) and the ESP-SPTC computed using the screened point charges (sc) for all atoms.

For all FMO calculations the Au atom was treated by using the triple-ζ model core potential (MCP-TZP) [36]. Therefore, hereafter, when a specific basis set is mentioned (e.g., 6-31G*) it is referred only to H, N, O, C, P and K atoms and the MCP-TZP for Au is always implied.

To summarize, we performed FMO calculations at the following levels of theory: FMO2 HF/6-31G*, FMO2 RI-MP2/6-31G, FMO2 RI-MP2/6-31G//sc, FMO2 RI-MP2/6-31G*, FMO2 RI-MP2/6-31G*//sc, FMO2 RI-MP2/6-311G, FMO2 RI-MP2/6-311G//sc, FMO3 RI-MP2/6-31G*, FMO3 RI-MP2/6-31G*//sc, FMO3 RI-MP2/6-311G//sc [37–40].

For each FMO calculation the water solvation effect was simulated through the PCM [1] method, by computing the repulsion and dispersion contributions by the empirical method of Floris and Tomasi [41], using a high density of tesserae on the cavity surface (NTSALL = 240) and FIXPVA as tessellation scheme [42]. The solvent screening effect was simulated using the partial screening method (MODPAR = 73) [43]. The cavities holding the solute were generated by adopting the simplified united atomic radii (radii = suahf). Charge compensation was included (ICOMP = 2) and cavitation energy was computed by Claverie-Pierotti method (ICAV = 1) at 298 K [44, 45].

To limit the computational burden, we performed the FMO3 calculations by adopting the low accuracy protocol (RESDIM = 2.5, RITRIM(1) = 0.001, −1, 1.25, 1.25).

When the screened point charges were adopted, the ESP-SPTC was computed using the charge damping [26] for all atoms with a = b = 1 (SCREEN = 1,1; RESPPC= −1).

Otherwise, the ESP-PTC approximation was only used between fragments with a VdW factor exceeding 4.00 (RESPPC = 4) and 2.50 (RESPPC = 2.5) for FMO2 and FMO3 calculations, respectively; for fragments separated by a distance smaller than the VdW factor, the ESP2 was used.

The energy error threshold for Pulay’s DIIS interpolation was set to 2.0 Hartree (ETHRSH = 2.0) and the density matrix convergence at which to switch from DIIS to second order SCF orbital optimization (SOSCF) was set to 0.005 (SWDIIS = 0.005). All FMO calculations included EDA and were performed by using the GAMESS-US package [46]. The DNA Gq structure was fragmented following the same scheme reported in the previous work [20]: DNA Gq was fragmented at N-glycosidic bond, N1–C1′, and at O–C5′ bond, to separate the nucleobase (guanine, G) and 2′-deoxyribose sugar and a phosphate group into distinct fragments, (Fig. S1) [47]. The covalent bond detachment was performed by using the hybrid orbital projection operator (HOP) scheme [48].

G10, G22 and the K^+^ ion (atom ID in 5CCW pdb file: K102) were considered as a unique fragment as well as G16, G4 and the other K^+^ atom (K101), as shown in Fig. S2. The FMO results were analyzed to evaluate *E*^*INT*^, along with the corresponding EDA, and to compute the FMO binding energies, *ΔE*^*FMO2*^ and *ΔE*^*FMO3*^.

## Results and discussion

### Ligand-receptor PIEs, E^INT^

The PIE between ligand and receptor fragments, *E*^*INT*^, is widely used in FMO LR study. In a previous work on Au(I)-biscarbene/Gq complexes [20], we experienced that the use of the most applied FMO computational settings, i.e., the FMO2 RI-MP2/6-31G* level of theory, leads to huge and unrealistically negative *E*^*INT*^ and *ΔE*^*FMO2*^ values when compared with experimental binding energy of − 10.4 kcal/mol (K_d_ = 0.03 µM) [26, 49]. There, we showed that this issue is related to the overestimation of the CT energy occurring when metal atoms are included in the target structure. We also found that, compared with local model, the partial screening method was necessary to properly account for the solvent screening effect and to improve the accuracy of *E*^*INT*^ of 1 and 2**.** Nevertheless, its adoption did not mitigate the overestimation of CT energy. In this section, to overcome this problem we analyse the impact of several factors, namely the level of theory, the basis set, the type of n-body approach and the use of screened point charges for all atoms in ESP-PTC calculation (ESP-SPTC), on *E*^*INT*^ values. The results are summarized in Table 1 and S1.

**Table 1:** *E*^*INT*^ values computed for Gq-1 and Gq-2 complexes at FMO2 RI-MP2/6-31G//sc, FMO2 RI-MP2/6-31G*//sc, FMO2 RI-MP2/6-311G//sc and FMO2 RI-MP2/6-311G//sc levels of theory. All values are in kcal/mol

Complex	FMO2 RI-MP2			FMO3 RI-MP2 *6-311G//sc*
	*6-31G//sc*	6-31G*//sc	*6-311G//sc*	
Gq(I)-1	− 31.6	− 36.5	− 63.1	− 60.5
Gq(II)-1	− 13.6	− 23.7	− 11.2	− 37.4
Gq(III)-1	− 24.8	− 34.9	− 38.2	− 52.6
average	− 23.3	− 34.9	− 45.7	− 50.2
Gq(I)-2	56.9	50.2	38.5	− 1.0
Gq(II)-2	− 19.3	– ^#^	− 29.6	− 39.0
Gq(III)-2	−^#^	− 30.872	− 36.2	− 50.6
average	18.8	5.4	− 12.9	− 30.2

^#^*E*^*INT*^ not computed due to unrecoverable issue with SCF convergence of some fragments

We preliminary evaluated the effect of the electron correlation comparing the *E*^*INT*^ values computed at FMO2 HF/6-31G* level of theory (Table S1) with those computed using the RI-MP2 method (FMO2 RI-MP2/6-31G*). Results show a relevant impact of correlation (Fig. S3A) which is important to correctly describe many-electrons system and to compute the dispersion interactions (*E*^*disp*^). Therefore, we evaluated the effect of the other computational features on E^INT^ only at RI-MP2 level of theory.

**Fig. 2:**
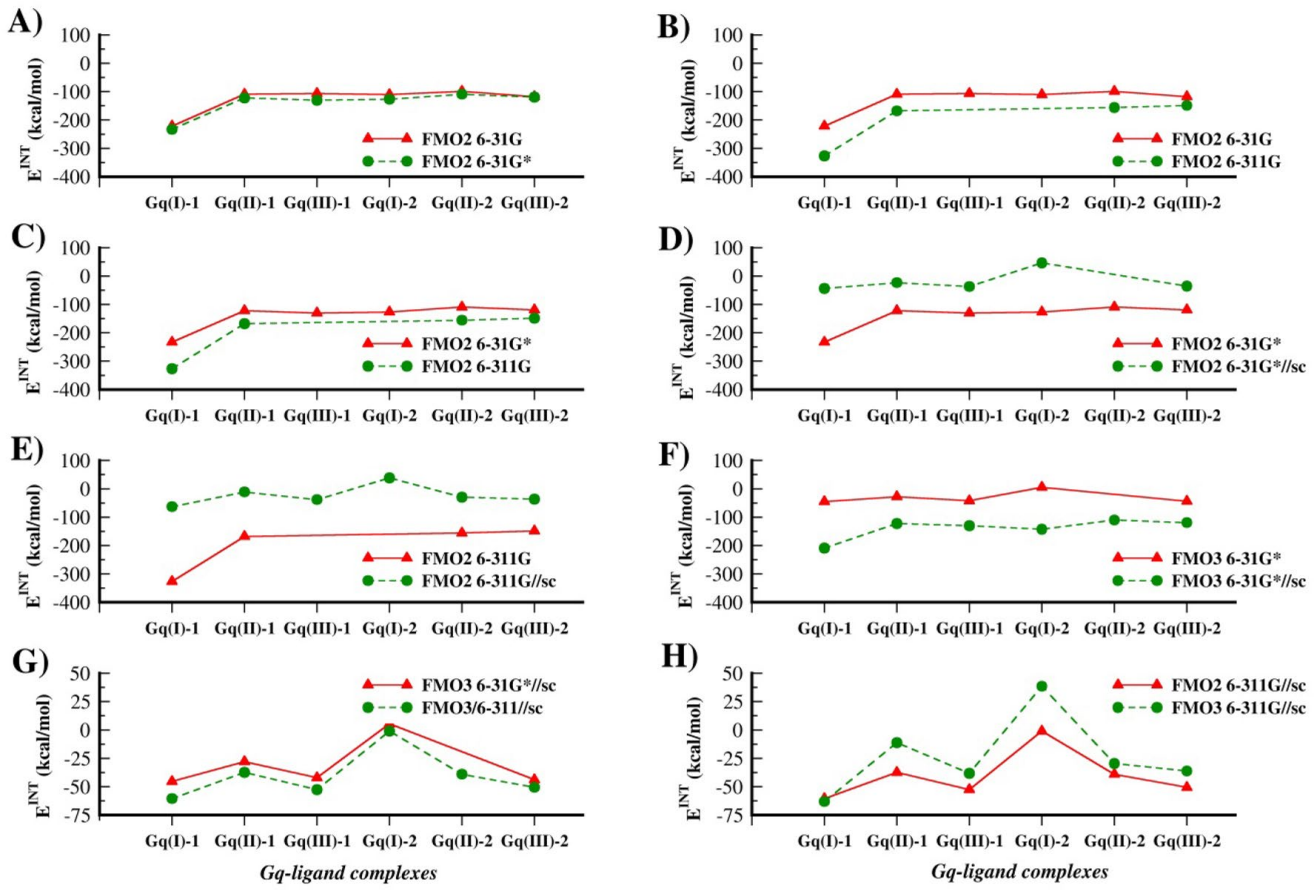
Comparison between *E*^*INT*^ values for ligands 1 and 2 at Gq(I), Gq(II) and Gq(III) binding sites, computed using different levels of theory: **a** FMO2 RI-MP2/6-31G vs. FMO2 RI-MP2/6-31G*; **b** FMO2 RI-MP2/6-31G vs. FMO2 RI-MP2/6-311G; **c** FMO2 RI-MP2/6-31G* vs. FMO2 RI-MP2/6-311G; **d** FMO2 RI-MP2/6-31G* vs. FMO2 RI-MP2/6-31G*//sc; **e** FMO2 RI-MP2/6-311G vs. FMO2 RI-MP2/6-311G//sc; **f** FMO3 RI-MP2/6-31G* vs. FMO3 RI-MP2/6-31G*//sc; **g** FMO3 RI-MP2/6-31G*//sc vs. FMO3 RI-MP2/6-311G//sc and **h** FMO2 RI-MP2/6-311G//sc vs. FMO3 RI-MP2/6-311G//sc. All energy values are reported in kcal/mol

The polarization (d) functions are important to increase the mathematical flexibility of the wave functions and to provide a better description of several chemical aspects (e.g., dipole moments, anions). To assess their impact, we compared the *E*^*INT*^ values computed with 6-31G and 6-31G* at RI-MP2 level of theory. As shown in Fig. 2a the inclusion of d functions determines a slight increase of the binding strength (more negative *E*^*INT*^) with no significant change in their magnitude.

The same effect, though to a larger extent, was found by expanding the basis set from 6-31G to 6-311G (Fig. 2b): *E*^*INT*^ terms become significantly more negative, even assuming an unrealistic value for Gq(I)-2 of −752.9 kcal/mol. The same conclusions can be obtained by comparing *E*^*INT*^ at 6-31G* and 6-311G at RI-MP2 level of theory (Fig. 2c).

Thus, the application of polarization d functions or the triple-ζ basis set (6-311G) does not sensibly improve the accuracy of *E*^*INT*^ which remains significantly more negative than experimental binding energy.

As mentioned in the *Theoretical background* section, the ESP can affect the charge transfer between *i* and *j* in dimer *ij*. In our previous work [20] the ESP was computed considering the point charge approximation (ESP-PTC) only between fragments exceeding the VdW factor 4.0 (RESPPC = 4.0) while the two electrons integral contribution was included for ESP between fragments below this value (ESP2). Notably, the ESP2 generally provides a more accurate description of ESP and therefore is generally the most applied approach [26].

We use here an alternative strategy in which the ESP-PTC is described by screened point charges for all atoms adopting the dumping function (ESP-SPTC), Eq. 8, setting a = b = 1, as suggested by the GAMESS-US manual. We also tried a = 1 and b = 2 but observed a negligible change in *E*^*FMO*^ and *E*^*INT*^ values as already reported by Fedorov et al. [26].

As shown in Fig. 2d, *E*^*INT*^ computed at FMO2 RI-MP2/6-31G*//sc are significantly smaller than the corresponding data found with FMO2 RI-MP2/6-31G* and the same results were found with 6-311G basis set (Fig. 2d). For instance, E^INT^ for Gq(I)-1 passes from − 326.6 to -36.5 kcal/mol, a more reasonable value closer to experimental reference data (− 10.4 kcal/mol). The relevant role in reducing the E^INT^ magnitude exerted by the ESP-SPTC is also observed for results obtained with the 6-31G basis set (Fig. S3B).

The use of the triple-ζ basis set leads to a slight increment of *E*^*INT*^ (Fig. 2e) but the value for Gq(I)-2 remains positive as found at FMO2 RI-MP2/6-31G*//sc level of theory.

The investigation of the complex between the Trp-cage protein bound with deprotonated p-phenolic acid highlights that the three-body approach corrects the overestimation of the CT energy in FMO2 and leads in general to more accurate PIE values [28]. However, we found that the application of the FMO3 approach alone was not enough to obtain more accurate E^INT^ values, confirming the requirement of the screened point charges in the ESP-PTC calculation, as shown in Fig. 2f.

Thus, while the binding affinity of ligand 1 is well described by *E*^*INT*^ computed at FMO2 RI-MP2/6-31G*//sc level of theory, for ligand 2 the best results have been obtained by employing the FMO3 approach with the 6-311G basis set (Fig. 2g), which determines more negative values of *E*^*INT*^ compared to those obtained with the 6-31G* basis set.

As shown in Fig. 2h, the *E*^*INT*^ calculated at FMO3 RI-MP2/6-311G//sc is characterized by the same trend found with FMO2 RI-MP2/6-311G//sc but with a significant improvement only for Gq(I)-2. Indeed, the corresponding *E*^*INT*^ is now negative (−1.0 kcal/mol), suggesting that the description of the interaction of ligand 2 at the position I requires a higher-level computational approach than FMO2 (Fig. 2g). Notably, the use of the FMO3 approach with low accuracy would require large basis set as 6-31G**, 6-311G*, 6-311G** (or larger) to produce results more accurate than those obtained with the FMO2 method. However, these basis sets significantly increase the computing time, making their application to routine CADD studies very difficult. Therefore, to assess the impact of the ESP-SPTC combined with such large basis sets using the FMO3 (low accuracy) we computed E^INT^ of ligand 1, with and without the screened point charges, considering a reduced model of the Gq receptor represented only by G nucleobases and K^+^ ions (Fig. S4). Results reported in Table 2 show that even with larger basis the FMO3 (low accuracy) alone does not provide accurate E^INT^ values. On the contrary, the application of ESP-SPTC allows obtaining more reliable results, closer to experimental binding energy.

**Table 2:** *E*^*INT*^ values computed for the reduced model of Gq(I)-1 (Fig. S4) using the FMO3 (low accuracy) method at RI-MP2/6-31G**, RI-MP2/6-311G*, RI-MP2/6-311G**, RI-MP2/6-31G**//sc, RI-MP2/6-311G*//sc and RI-MP2/6-311G**//sc levels of theory. All values are in kcal/mol

Level of theory	FMO3 E^INT^reduced Gq(I)-1 complex
RI-MP2/6-31G**	− 228.4
RI-MP2/6-311G*	− 299.9
RI-MP2/6-311G**	− 302.1
RI-MP2/6-31G**//sc	− 48.4
RI-MP2/6-311G*//sc	− 52.3
RI-MP2/6-311G**//sc	− 54.3

**Fig. 3:**
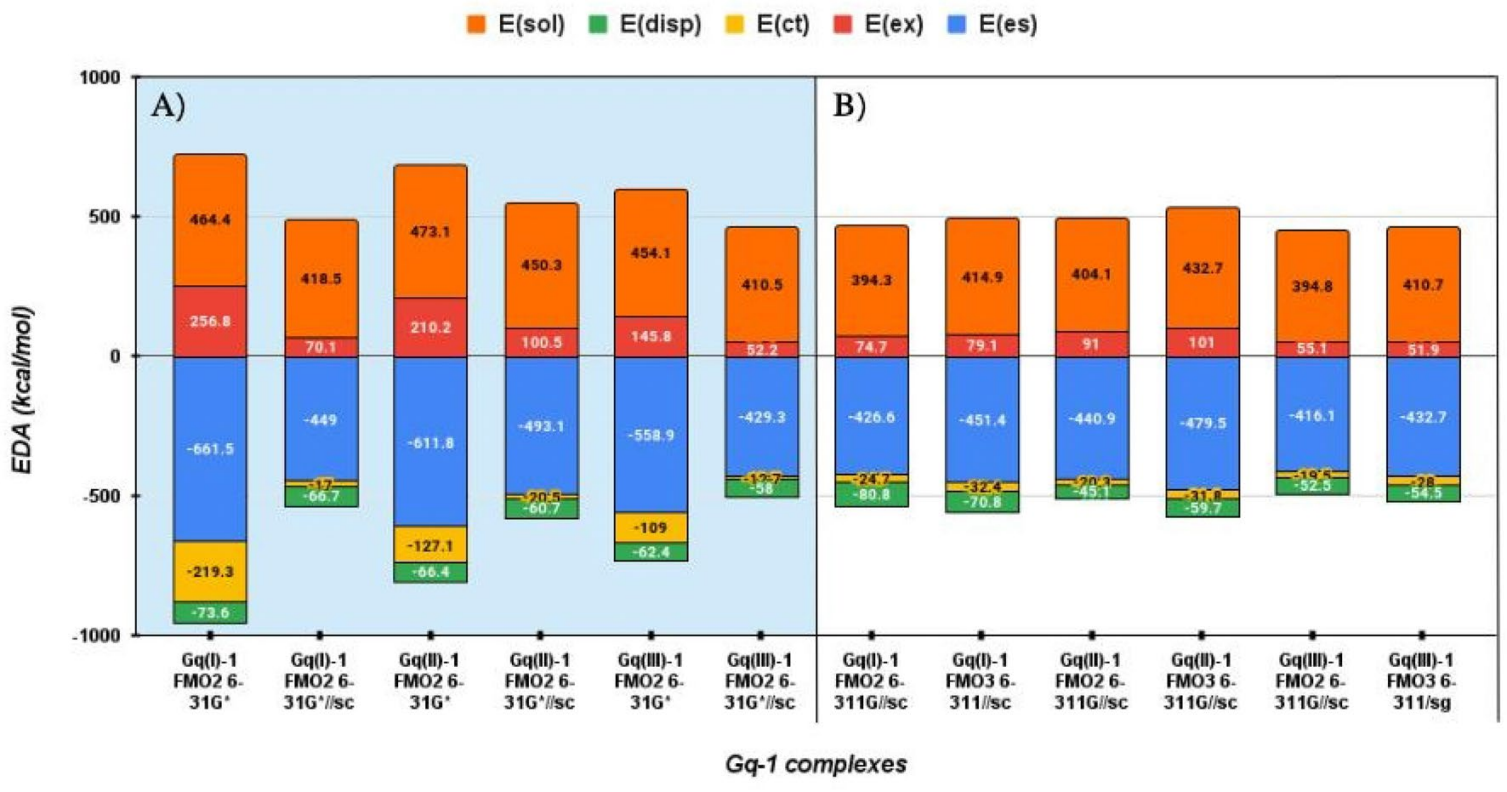
Bar diagram of total PIEDA for Gq-1, considering the three different binding regions I, II and III, computed at the FMO2 RI-MP2/6-31G*, FMO2 RI-MP2/6-31G*//sc, FMO2 RI-MP2/6-311G//sc and FMO3 RI-MP2/6-311G//sc levels of theory. *E*^*es*^, *E*^*ex*^, *E*^*ct*^, *E*^*disp*^ and *E*^*sol*^ are the electrostatic, exchange repulsion, charge transfer, dispersion and solvation energies, respectively

The EDA of *E*^*INT*^ provides a crucial insight on the nature of bonding and allows monitoring how the contribution of CT energy varies as a function of the basis set magnitude and upon the use of the ESP-SPTC. In Fig. 3 and in Table S2 we reported the EDA for ligand 1 at the three binding poses while the corresponding values for ligand 2 are reported in Fig. S5 and Table S3.

Figure 3a shows the impact of the ESP-SPTC on the EDA terms at the FMO2 RI-MP2/6-31G* level of theory. *E*^*es*^, *E*^*ex*^ and *E*^*ct*^ are significantly influenced by the screened point charges that markedly decrease the absolute value of these energy contributions. *E*^*ct*^ is the most affected term passing from −219.3, −127.1 and −109.0 to −17.0, −20.5 and 19.7 kcal/mol for Gq(I)-1, Gq(II)-1 and Gq(III)-1, respectively. This evidence highlights the crucial contribution of the ESP-SPTC to provide correct estimates of the *E*^*ct*^ term.

The use of the 6-311G basis set and of FMO3 approach leads in general to more negative *E*^*INT*^ values, with relative weights of *E*^*es*^, *E*^*ex*^, *E*^*ct*^, *E*^*disp*^ and *E*^*solv*^ contributions that resemble those obtained at FMO2 RI-MP2 6-31G*//sc (Fig. 3b).

A similar situation is found for the EDA of *E*^*INT*^ performed for ligand 2 (Fig. S5 and Table S3). In this case, as mentioned before, the FMO3 treatment results to be crucial to correctly estimate the attractive contribution of *E*^*ct*^ (−22.8 kcal/mol) in Gq(I)-2 complex, even with the 6-311G basis set, for which we found a positive value of +14.2 kcal/mol.

**Fig. 4:**
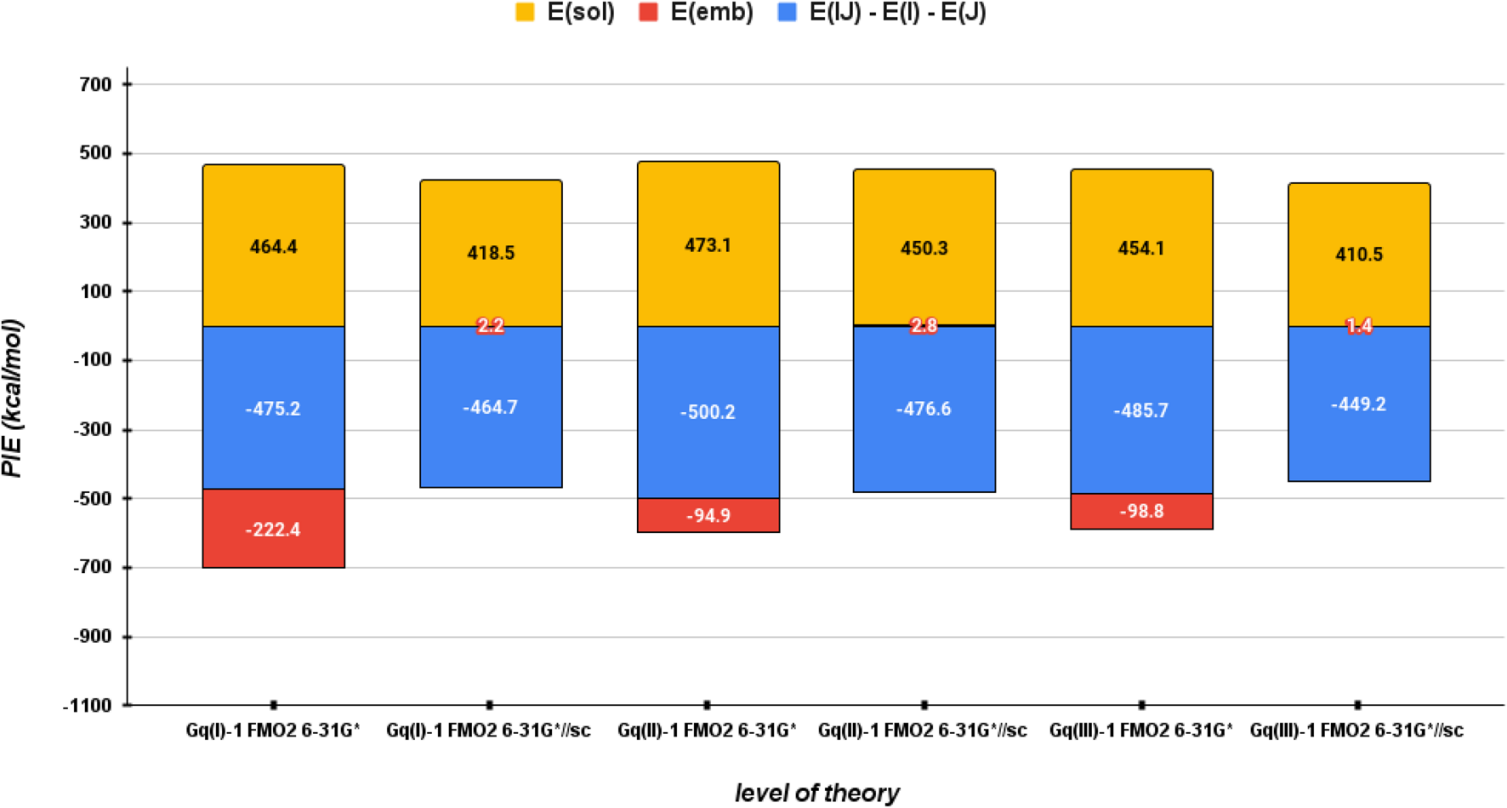
Bar diagram of *E*^*INT*^ decomposed according to Eq. 15 for Gq-1, considering the three different binding regions I, II and III, at the FMO2 RI-MP2/6-31G* and FMO2 RI-MP2/6-31G*//sc levels of theory. *E*^*sol*^, *E*^*emb*^ and (*E*_*Li*_ - *E*_*L*_ - *E*_*i*_) are the solvation energies, the embedding energy (that is the sum of ligand-receptor embedded charge transfer energies) and the energy difference between the internal solute energy respects with Li complex, respectively

According to Eq. 15, *E*^*INT*^ can be decomposed into three terms,$\sum _{i=1}^{N}({E}_{Li}^{{\prime }{\prime }}- {E}_{i}^{{\prime }{\prime }}-{E}_{L}^{{\prime }{\prime }})$, $\sum _{i=1}^{N}{E}_{Li}^{sol}$ and $\sum _{i=1}^{N}{E}_{Li}^{emb}$ where the latter term is the embedded charge transfer energy (*Tr(ΔD*^ij^*V^*ij*^*)*) between ligand and all Gq fragments. As shown in Fig. 4 and reported in Table S4, *E*^*emb*^ is the energy term that changes most by applying the screened point charges for all atoms, from large negative (attractive) to small positive values. Indeed, passing from FMO2 RI-MP2/6-31G* to FMO2 RI-MP2/6-31G*//sc level of theory this term changes from −222.4, −94.9 and −98.8 to 2.2, 2.8 and 1.4 kcal/mol for Gq(I)-1, Gq(II)-1 and Gq(III)-1, respectively, confirming that the ESP-SPTC is crucial to fix the embedded CT overestimation of pair interactions involving metals-containing fragments.

**Fig. 5:**
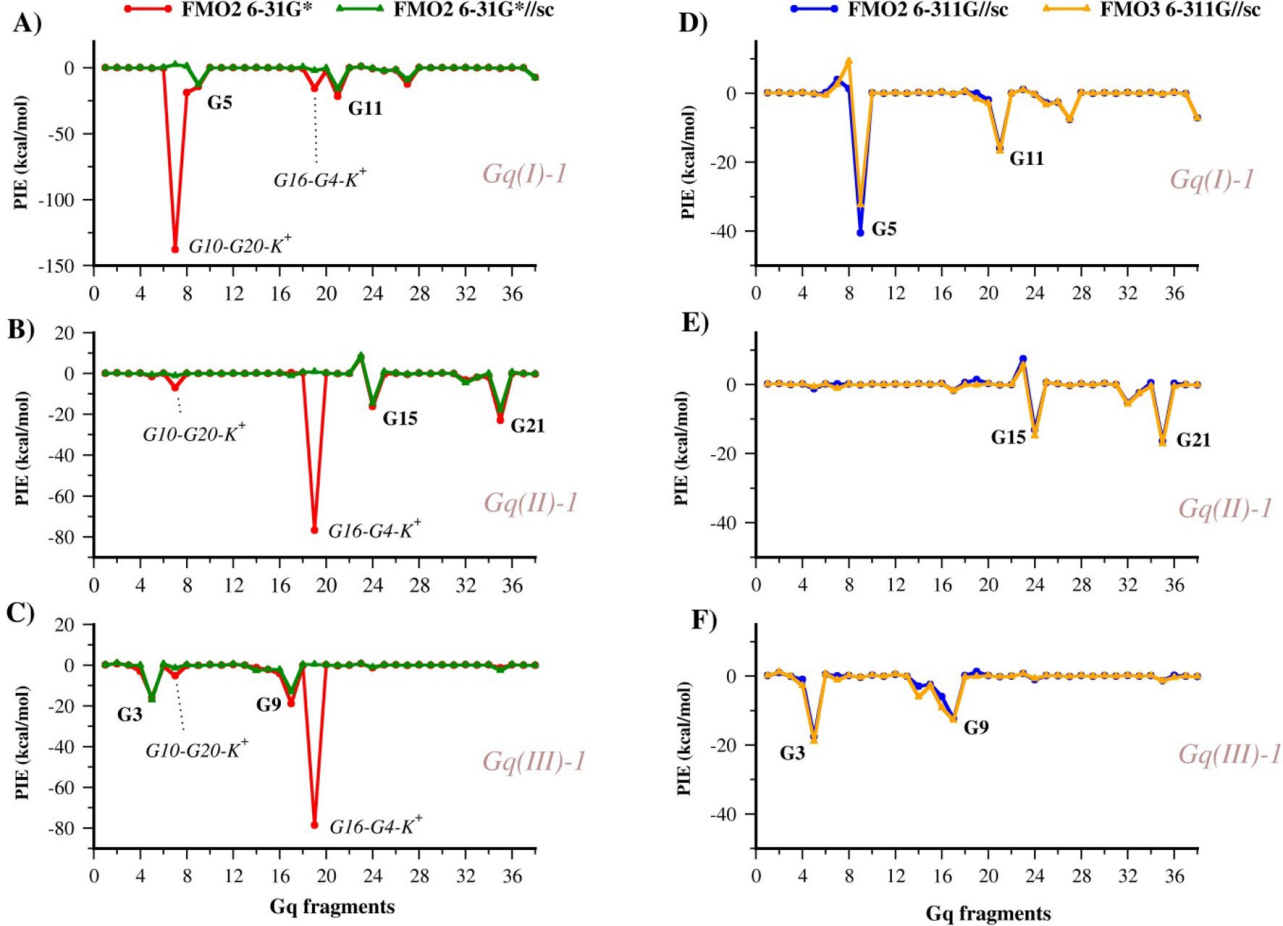
${E}_{Li}^{PIE}$ values for the interaction between Gq fragments and ligand 1 in the binding sites I (**A** and **D**), II (**B** and **E**) and III (**C** and **F**), computed at the FMO2 RI-MP2/6-31G*, FMO2 RI-MP2/6-31G*//sc, FMO2 RI-MP2/6-311G//sc and FMO3 RI-MP2/6-311G//sc levels of theory and reported by using red, green, blue and orange lines, respectively

Ligand 1 with the nearest fragments containing K^+^, i.e., fragments 7 and 19 for Gq(I) and Gq(II)/Gq(III), respectively, shows the most significant *E*^*ct*^ decrease, with a consequent improvement of the *E*^*INT*^ accuracy, as shown by diagrams reported in Fig. 5a, b and c. The PIE of fragment 7-ligand 1 calculated with the 6-31G* basis set is −137.9 kcal/mol which becomes 4.0, 2.5 and 2.6 kcal/mol by adopting the screened point charges for all atoms at FMO2 RI-MP2/6-31G*, FMO2 RI-MP2/6-311G and FMO3 RI-MP2/6-311G levels of theory (Fig. 5d, e and f and Table S5), respectively. As reported in our previous work (Table S2 of ref. 19), the *E*^*es*^ contributions to the PIE between the two positively charged fragments, i.e., 1 and fragment 7/fragment 19 in Gq(I) computed at FMO2 RI-MP2 6-31G*, are negative values, leading to some inconsistency. On the contrary, the inclusion of the screened point charges yields positive *E*^*es*^ values, correctly indicating a repulsive electrostatic interaction between two fragments with the same charge. All other energy terms, except *E*^*solv*^, become negligible with values close to zero.

As reported in Table S6, the same favourable effects of ESP-SPTC on *E*^*es*^ and *E*^*ct*^ terms were observed in PIEs between 1 and (G22∙G10∙K102) fragment computed for reduced Gq(I)-1 complex with FMO3 (low accuracy) using large basis set (6-31G**, 6-311G* and 6-311G**).

Notably, the impact of ESP-SPTC can be also appreciated by considering amount of CT, Q^CT^, from ligand to the nearest fragment containing K^+^ ion (Table S5-S7). Indeed, for instance, for Gq(I)-1 the Q^CT^ are −0.2095 and −0.0126 at FMO2 RI-MP2/6-31G* and FMO2 RI-MP2/6-31G*//sc, respectively, suggesting that ESP-SPTC might effectively reduce the overestimation of CT. The analysis of other Q^CT^ values confirms this trend.

Comparable results are also found for ligand 2 (Fig. S6, Table S3 and S7), although a positive value of *E*^*INT*^ is obtained for Gq(I)-2 at FMO2 RI-MP2/6-31G*//sc.

By applying the ESP-SPTC, the profile of PIEs chart is significantly simplified where the most relevant interactions are limited to those between the ligand and the underlying nucleobases (Fig. 5), which are G5–G11, G15–G21 and G3–G9 at Gq(I), Gq(II) and Gq(III) binding sites, respectively.

As shown in Fig. 3, the main contribution to *E*^*INT*^ is represented by electrostatic energy, with a lower contribution of *E*^*disp*^ and *E*^*ct*^. These two terms are significative only in the pair interactions between ligands and the underlying nucleobases DG5, DG11 DG15, DG21, DG9 and DG3 (Table S8) which are the closest to the ligands. The EDA of the interactions between the ligand and these nucleobase pairs (Tables S8 and S9) confirms the asymmetric interaction of ligands 1 and 2 as reported in the previous work [20], supporting the occurrence of π-cation interaction hypothesized for 1.

The average PIEs between ligands and the underlying guanines pairs are in good agreement with the average *E*^*INT*^ values (Table S10) suggesting that this specific interaction energy can be used to quickly assess the binding affinity of a set of Gq binders, especially if the FMO method is combined with either molecular dynamics or other multi-conformational approaches where the execution of many FMO calculations is needed.

The average *E*^*INT*^ values defined for Gq-1 and Gq-2 complexes are −50.2 and −30.2 kcal/mol, respectively, at FMO3 RI-MP2/6-311G//sc level of theory and −45.7 and − 12.9 kcal/mol at FMO2 RI-MP2/6-311G//sc, well reproducing the trend of the experimental binding efficiency of two Au(I)-binders investigated in this work (Table 2). For ligand 1, the average *E*^*INT*^ value closest to experimental binding energy (−10.4 kcal mol^−1^) was instead computed at FMO2 RI-MP2/6-31G*//sc level of theory (−34.9 kcal/mol).

The present results indicate that the use of the EPS-SPTC should be considered to correctly describe the CT energy in a peculiar system as the Au(I)-biscarbene/Gq complexes. This evidence agrees with the methodological study of water clusters performed by Fedorov et al. [26] where a worse performance of ESP2 compared to ESP-PTC was found, the more so with the increasing of basis set size. The same effect is shown here when passing from double-ζ to triple-ζ basis sets. ESP2 can promote the convergence to an unphysical electronic state with consequent abnormal CT between the two fragments [20, 26]. Finally, again in agreement with the results of Fedorov et al. [26], the relevant improvement in the accuracy produced by the charges’ screening can be explained with their capability to significantly reduce the polarization of the electronic state at short distance [26]. However, for common applications the default EPS-PTC/ESP2 approach should always be considered as the reference method.

As a final remark, it is worth noting that Gq/Au(I)-biscarbene complexes represent a challenging system from the computational point of view where two K^+^ ions are in a channel surrounded by negatively charged sugar-phosphate skeleton and the positively charged Au(I) ligands are located perpendicular to the vertical axis connecting the two K^+^ ions. This peculiar geometrical configuration, where the distance between ligands and nearest K^+^ ions is 5–6 Å (Fig. S7), might promote charge transfer, making the correct evaluation of the related energy difficult.

### FMO binding energy, ΔE^FMO^

The *ΔE*^*FMO*^, conversely from *E*^*INT*^, considers the polarization-destabilization and desolvation energies of both ligand and receptor passing from free to bound state, providing in principle a better description of the binding process.

According to Eq. 1, *ΔE*^*FMO*^ is strictly related to *E*^*INT*^. Therefore, we computed *ΔE*^*FMO*^ by using the best computational settings found for *E*^*INT*^ such as the FMO2 RI-MP2/6-31G* and FMO3 RI-MP2/6-311G levels of theory combined with the ESP-SPTC.

As shown in Table S11, the use of screened charges for all atoms has a huge effect on *ΔE*^*FMO*^ values calculated at FMO2 RI-MP2/6-31G* and FMO3 RI-MP2/6-311G levels of theory, significantly reducing their magnitude as find for *E*^*INT*^. Both FMO2 and FMO3 binding energies computed with ESP-SPTC are positive values in the most cases, hampering a direct comparison with experimental binding data. This result might also suggest that the adoption of the ESP-SPTC for all atoms might lead to a possible slight underestimation of interaction energies. The employ of a large basis set, facilitated by the auxiliary polarization, FMO/AP [50], might improve the accuracy of *ΔE*^*FMO*^ values computed using the ESP-SPTC.

It should be remarked that *ΔE*^*FMO*^ does not include entropy that could give a crucial contribution to the binding, especially when hydration entropy is considered. In the formation of the LR complex, both isolated ligand and receptor release the hydration water molecules with subsequent increase of entropy. The hydration entropy was indeed found to be crucial to determine negative energies for the binding of small peptides to RNA quadruplex [51]. However, considering that ligands 1 and 2 are small molecules compared with a peptide, the real contribution to the hydration entropy for biscarbene-Au(I)/Gq binding process should be carefully evaluated and could not be enough to compensate the positive *ΔE*^*FMO*^ value.

In the present case, where no entropic contributions were considered, the *E*^*INT*^ values computed with the ESP-SPTC show a better agreement with experimental evidence than *ΔE*^*FMO*^ values, even at FMO2 level, representing therefore the best term to describe the binding affinity of metal-containing ligands to DNA-Gq. The description of systems like biscarbene-Au(I)/Gq might also benefit from the application of partition analysis method based on charge transfer state with fractional charges proposed for the DFTB [52].

In summary, the computational protocol to assess the binding efficiency of metal-binders to DNAGq by adopting the FMO method should include (i) the partial screening method to correctly simulate the solvent screening effect and should consider the application of (ii) the ESP-SPTC for all atoms when questionable PIE values are obtained using the default ESP treatment. Combined with these features, the FMO2 RI-MP2/6-31G* level of theory provides satisfactory results for *E*^*INT*^ while the adoption of larger basis set might improve the accuracy when it is coupled with FMO3 approach. However, we suggest applying the three-body approach only in critical cases where the FMO2 method does not ensure enough accuracy, like structures containing many charged fragments close to each other.

It is finally worth noting that the accuracy in the prediction of a set of ligands binding affinity based on *E*^*INT*^ can be significantly improved by its combination with additional energy terms, e.g., entropic and hydrophobic terms. A recent study, where *E*^*INT*^ was linearly combined with clogP, demonstrated that a similar approach can be effectively used to predict with great accuracy (R^2^ = 0.9) the binding efficiency of ligand-receptor systems not containing metal atoms [53]. Our future work will be devoted to developing a scoring function based on our FMO/GRID approach [7, 10] specifically designed to predict the binding energy of LR complexes containing metals.

## Conclusions

Metal atoms are widely present in biological macromolecules and often play a critical role on their reactivity or structural stability, as is the case of Gq motif. In this system, the quadruplex motif is shaped by the presence of K^+^ ions inducing electrostatic, polarization and charge transfer phenomena that can be described with accuracy only by using QM methods. The identification of Gq structures in oncogenes has prompted the evaluation of small molecules able to hit this peculiar DNA motif as a new promising approach for cancer treatment. Au(I)-biscarbene derivatives, as 1 and 2, showed a significant binding affinity for Gq. In a previous work, we computed their *ΔE*^*FMO2*^ and *E*^*INT*^ of Gq-1 and Gq-2 complexes showing that ab initio FMO2 method at RI-MP2 6-31G*//PCM [1] level of theory, the usual approach in FMO2 ligand-receptor studies, led to huge negative values due to the overestimation of the embedded charge transfer energy between fragments containing metal atoms. This issue hampers the comparison with experimental binding energy values and limits the usefulness of the FMO in CADD study for systems containing metal. To overcome this problem and enhance the accuracy of the FMO method for predicting the binding affinity of metal-based ligands interacting with Gq, in the present work we evaluated the impact of four computational aspects on the FMO results: *(i)* electron correlation, by considering the Hartree–Fock (HF) and a post-HF method as RI-MP2; *(ii)* two- (FMO2) versus three-body (FMO3) approaches; *(iii)* the basis set type (inclusion of the polarization functions and double-ζ vs. triple-ζ basis sets) and *(iv)* the use of screened point charges for all atoms in the ESP-PCT computation (ESP-SPTC). Moreover, the partial screening method was systematically adopted for each calculation.

We found that, although the ESP-SPTC for all atoms provides in general a less accurate description of ESP, it has the most relevant impact on the *E*^*INT*^ and *ΔE*^*FMO*^ leading to values with magnitude comparable to experimental binding energy.

Indeed, its use completely removes the overestimation of the CT energy. Our results suggest that the best computational settings for FMO calculation at RI-MP2 level of theory for Au(I)-biscarbene/Gq complexes should include:

(i) The partial screening method to simulate the screening effect of the solvent.
(ii) The adoption of the ESP-PTC computed using the screened point charges for all atoms.

Though *E*^*INT*^ values do not include the destabilization polarization and desolvation energies, we found that this term is a better descriptor of the binding efficiency trend of Gq-1 and Gq-2 than *ΔE*^*FMO*^, using the FMO2 method and the 6-31G* basis set (or larger) combined with above-mentioned features. Moreover, *E*^*INT*^ can be easily combined with other energy terms, as the entropic and hydrophobic contributions, leading to an enhanced prediction of the binding affinity with a lower computational effort than *ΔE*^*FMO*^.

We envision that the computational protocol described in this work should be considered for FMO calculations regarding biological systems including metals, where CT phenomena are widely present, and the adoption of the default ESP treatment entails a misleading evaluation of pair interaction energies.

### Supplementary information

Below is the link to the electronic supplementary material.

Supplementary material 1 (PDF 1030.8 kb)

## Data Availability

The 3D coordinates of Gq, Gq-1_3_, Gq-1(I), Gq-1(II), Gq-1(III), Gq-2(I), Gq-1(II), Gq-2(III), ligands **1** and **2** structures used this study are available in the pdb and xyz file formats at the following link 10.5281/zenodo.7102260 .

## Abbreviations

CADD: Computer assisted drug discovery
CT: Charge transfer
EDA: Energy decomposition analysis
ESP: Embedding electrostatic potential
ESP: Embedding electrostatic potential
ESP2, ESP: Computed considering the two-electron integral contributions
ESP-PTC, ESP: Computed by using Mulliken point charges
ESP-SPTC, ESP: Computed by using the screened Mulliken point charges for all atoms
FMO: Fragment molecular method
FMO2: Two-body FMO approach
FMO3: Three-body FMO approach
Gq: DNA G-quadruplex structure
Gq(I): Binding site on DNA Gq motif (PDB ID: 5CCW) delimited by G5·G11 guanine pair
Gq(II): Binding site on DNA Gq motif (PDB ID: 5CCW) delimited by G15·G21 guanine pair
Gq(III): Binding site on DNA Gq motif (PDB ID: 5CCW) delimited by G3·G9 guanine pair
HIE: Hydrophobic interaction energy
MM: Molecular mechanics
PIE: Pair interaction energy
QM: Quantum mechanics
