# Supplementary material for: Improving the accuracy of the FMO binding affinity prediction of ligand-receptor complexes containing metals
Source: J Comput Aided Mol Des. 2023 Sep 25;37(12):707–19. doi: 10.1007/s10822-023-00532-2 (PMC10618332; doi:10.1007/s10822-023-00532-2)
Supplement: Supplementary file 1 — Supplementary material 1 (PDF 1030.8 kb) [file 10822_2023_532_MOESM1_ESM.pdf]

Supporting Information

Improving the accuracy of the FMO binding affinity prediction of ligand-receptor complexes containing metals

R. Paciotti[1]*, A. Marrone[1], C. Coletti[1], N. Re[1]

[1] Department of Pharmacy, Università "G. D'Annunzio" Di Chieti-Pescara, Chieti, Italy

*corresponding to: r.paciotti@unich.it

Table of Contents

Fig. S1 2

Fig. S2 2

Table S1 3

Fig. S3 3

Fig. S4 4

Table S2 5

Fig. S5 5

Table S3 6

Table S4 6

Fig. S6 7

Table S5 8

Table S6 9

Table S7 10

Table S8 11

Table S9 12

Table S10 13

Fig. S7 14

Table S11 14

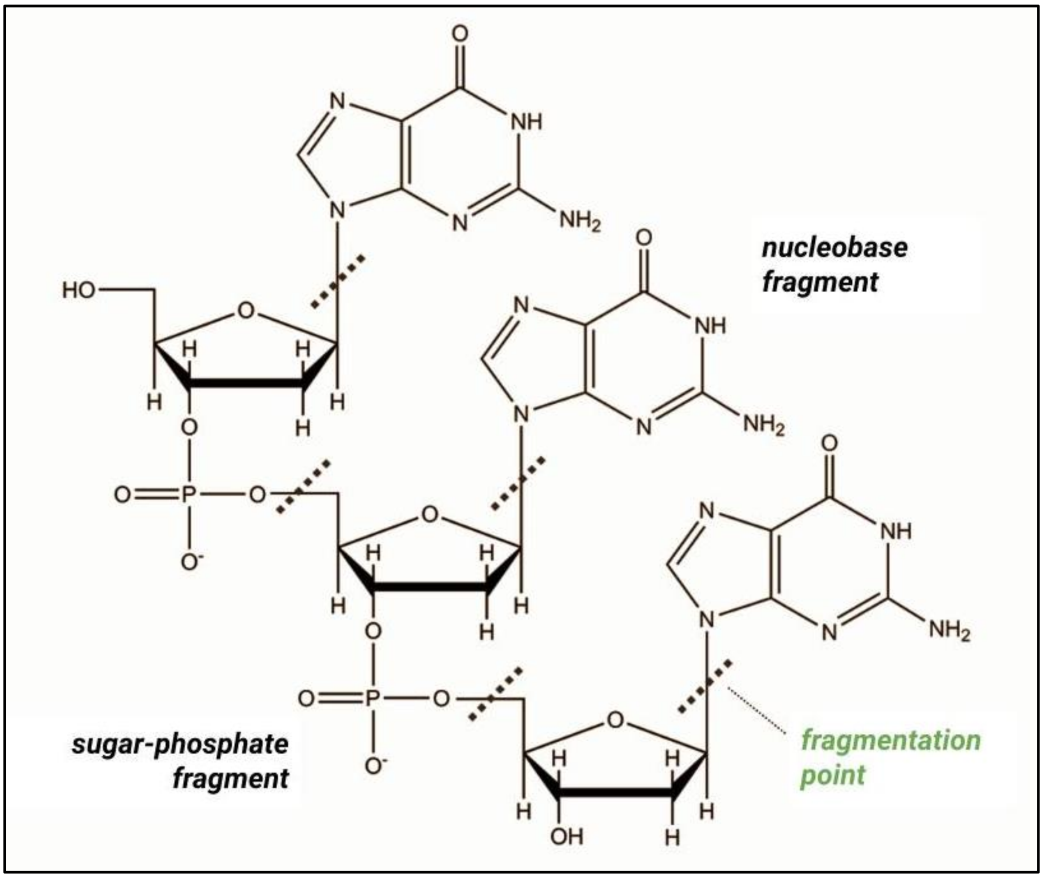

Fig. S1 Fragmentation scheme adopted in this work. Two types of fragments are obtained: sugar-phosphate and nucleobase fragments. The fragmentation point is represented by a dashed line

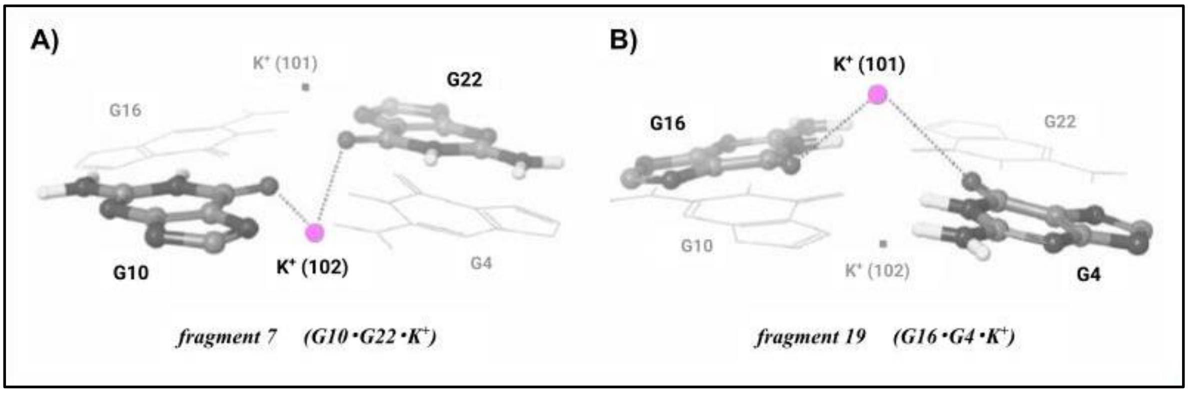

Fig. S2 a) *fragment 7* including G10, G22 and K^+ ions (K102); b) *fragment 19* including G16, G4 and K+ ions (K101)

Table S1 E^{INT} values computed for **Gq-1** and **Gq-2** complexes at FMO2 HF/6-31G*, FMO2 RI-MP2/6-31G, FMO2 RI-MP2/6-31G*, FMO2 RI-MP2/6-311G, FMO3 RI-MP2/6-31G* and FMO3 RI-MP2/6-31G*//sc levels of theory. All values are in kcal/mol

**Complex**	**FMO2 HF/6-31G***	**FMO2 RI-MP2**			**FMO3 RI-MP2**	
		6-31G	***6-31G****	***6-311G***	***6-31G****	***6-31G*//sc***
Gq(I)-1	-159.6	-221.8	-233.2	-326.6	-208.9	-45.4
Gq(II)-1	-55.5	-109.0	-122	-168	-122.2	-28
Gq(III)-1	-68	-106.8	-130.4	- #	-130.1	-42.1
average	**-94.4**	**-145.9**	**-161.9**	**-247.3**	**-153.7**	**-38.5**
Gq(I)-2	-52.86	-110.4	-127.1	-752.9	-142.4	5.4
Gq(II)-2	-48.9	-98.8	-109.2	-155.9	-110.2	- #
Gq(III)-2	-60.9	-117.5	-119.2	-148.7	-119.3	-43.8
average	**-54.2**	**-108.9**	**-118.5**	**-352.5**	**-124.0**	**-19.2**

E^{INT} not computed due to unrecoverable issue with SCF convergence of some fragments

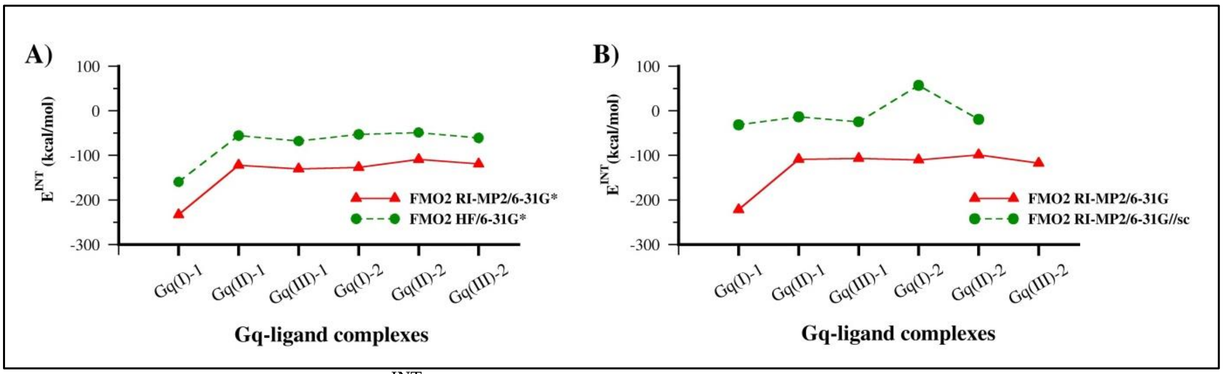

Fig. S3 Comparison between E^{INT} values related to ligands **1** and **2** at Gq(I), Gq(II) and Gq(III) binding sites, computed at a) FMO2 HF/6-31G* (red line) and FMO2 RI-MP2/6-31G* (green line) levels of theory and at b) FMO2 RI-MP2/6-31G (red line) and FMO2 RI-MP2/6-31G//sc (green line) levels of theory. All energy values are reported in kcal/mol

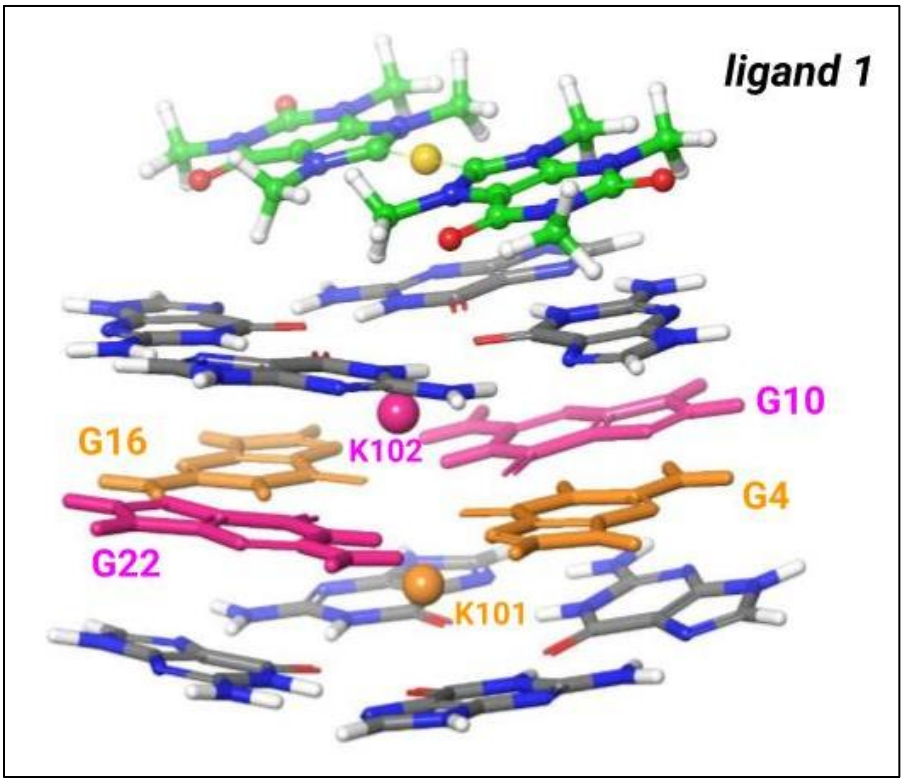

Fig. S4 Reduced model of Gq(I)-1 complex where Gq receptor is represented only by G nucleobases and K^+ ions. This model was used to perform FMO3 (low accuracy) calculations at RI-MP2 level of theory using the 6-31G**, 6-311G* and 6-311G** basis sets. The same fragmentation scheme adopted for entire Gq structure was used. The fragments including K^+ (101), G4 and G16 (orange) and K^+ (102), G10 and G22 (pink) correspond to fragments 19 and 7, respectively, of the entire Gq(I)-1 complex (Fig. S2)

Table S2 EDA of E^{INT} considering ligand **1** at I, II and III binding sites, computed at the FMO2 RI-MP2/6-31G*, FMO2 RI-MP2/6-31G*//sc, FMO2 RI-MP2/6-311G//sc and FMO3 RI-MP2/6-311G//sc levels of theory. All energy values are reported in kcal/mol

Complex – level of theory	EDA of E^{INT}				
	E^{es}	E^{ex}	E^{ct}	E^{disp}	E^{sol}
Gq(I)-1 – FMO2 6-31G*	-661.5	256.8	-219.3	-73.6	464.4
Gq(I)-1 – FMO2 6-31G*//sc	-449.0	70.1	-17.0	-66.7	418.5
Gq(II)-1 – FMO2 6-31G*	-611.8	210.2	-127.1	-66.4	473.1
Gq(II)-1 – FMO2 6-31G*//sc	-493.1	100.5	-20.5	-60.7	450.3
Gq(III)-1 – FMO2 6-31G*	-558.9	145.8	-109.0	-62.4	454.1
Gq(III)-1 – FMO2 6-31G*//sc	-429.3	52.2	-12.7	-58.0	410.5
Gq(I)-1 – FMO2 6-311G//sc	-426.6	74.7	-24.7	-80.8	394.3
Gq(I)-1 – FMO3 6-311//sc	-451.4	79.1	-32.4	-70.8	414.9
Gq(II)-1 – FMO2 6-311G//sc	-440.9	91.0	-20.3	-45.1	404.1
Gq(II)-1 – FMO3 6-311G//sc	-479.5	101.0	-31.8	-59.7	432.7
Gq(III)-1 – FMO2 6-311G//sc	-416.1	55.1	-19.5	-52.5	394.8
Gq(III)- – FMO3 6-311/sg	-432.7	51.9	-28.0	-54.5	410.7

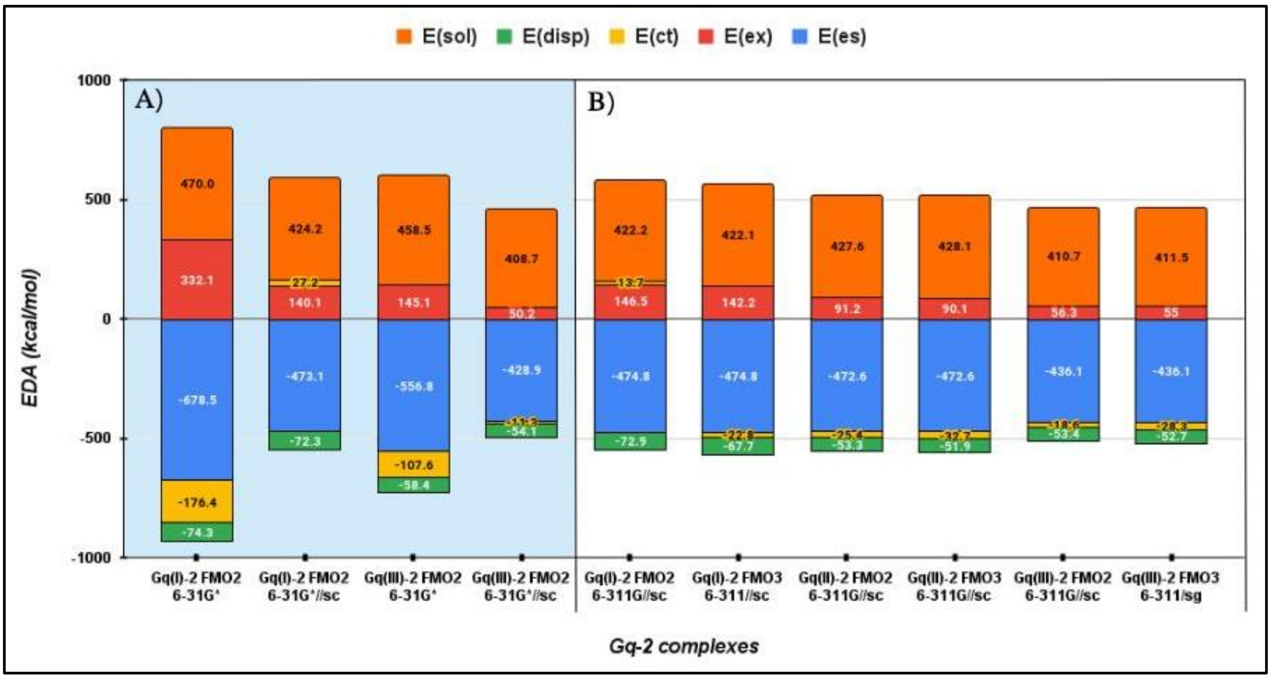

Fig. S5 Bar diagram of total PIEDA for **Gq-2**, considering the three different binding regions I, II and III, computed at the FMO2 RI-MP2/6-31G*, FMO2 RI-MP2/6-31G*//sc, FMO2 RI-MP2/6-311G//sc and FMO3 RI-MP2/6-311G//sc levels of theory. E^{es}, E^{ex}, E^{ct}, E^{disp} and E^{sol} are the electrostatic, exchange repulsion, charge transfer, dispersion and solvation energies, respectively

Table S3 EDA of E^{INT} considering ligand **2** at I, II and III binding sites, computed at the FMO2 RI-MP2/6-31G*, FMO2 RI-MP2/6-31G*//sc, FMO2 RI-MP2/6-311G//sc and FMO3 RI-MP2/6-311G//sc level of theories. All energy values are reported in kcal/mol

**Complex – level of theory**	**EDA of E^{INT}**				
	**E^{es}**	**E^{ex}**	**E^{ct}**	**E^{disp}**	**E^{sol}**
Gq(I)-2 – FMO2 6-31G*	-678.5	332.1	-176.4	-74.3	470.0
Gq(I)-2 – FMO2 6-31G*//sc	-473.1	140.1	27.2	-72.3	424.2
Gq(III)-2 – FMO2 6-31G*	-556.8	145.1	-107.6	-58.4	458.5
Gq(III)-2 – FMO2 6-31G*//sc	-428.9	50.2	-11.3	-54.1	408.7
Gq(I)-2 – FMO2 6-311G//sc	-474.8	146.5	13.7	-72.9	422.2
Gq(I)-2 – FMO3 6-311//sc	-474.8	142.2	-22.8	-67.7	422.1
Gq(II)-2 – FMO2 6-311G//sc	-472.6	91.2	-25.4	-53.3	427.6
Gq(II)-2 – FMO3 6-311G//sc	-472.6	90.1	-32.7	-51.9	428.1
Gq(III)-2 – FMO2 6-311G//sc	-436.1	56.3	-18.6	-53.4	410.7
Gq(III)-2 – FMO3 6-311/sc	-436.1	55	-28.3	-52.7	411.5

Table S4 Decomposition of E^{INT} values according to eq. 16, computed for Gq-1 complexes, considering the I, II and III binding sites using the FMO2 6-31G* and FMO2 6-31G*//sc approaches. All energy values are in kcal/mol

**Complex – level of theory**	**E^{INT} decomposition (eq. 12)**		
	**$\sum(E_{Li} - E_i - E_L)$**	**$\sum E^{emb}$**	**E^{sol}**
Gq(I)-1 FMO2 6-31G*	-475.2	-222.4	464.4
Gq(I)-1 FMO2 6-31G*//sc	-464.7	2.2	418.5
Gq(II)-1 FMO2 6-31G*	-500.2	-94.9	473.1
Gq(II)-1 FMO2 6-31G*//sc	-476.6	2.8	450.3
Gq(III)-1 FMO2 6-31G*	-485.7	-98.8	454.1
Gq(III)-1 FMO2 6-31G*//sc	-449.2	1.4	410.5

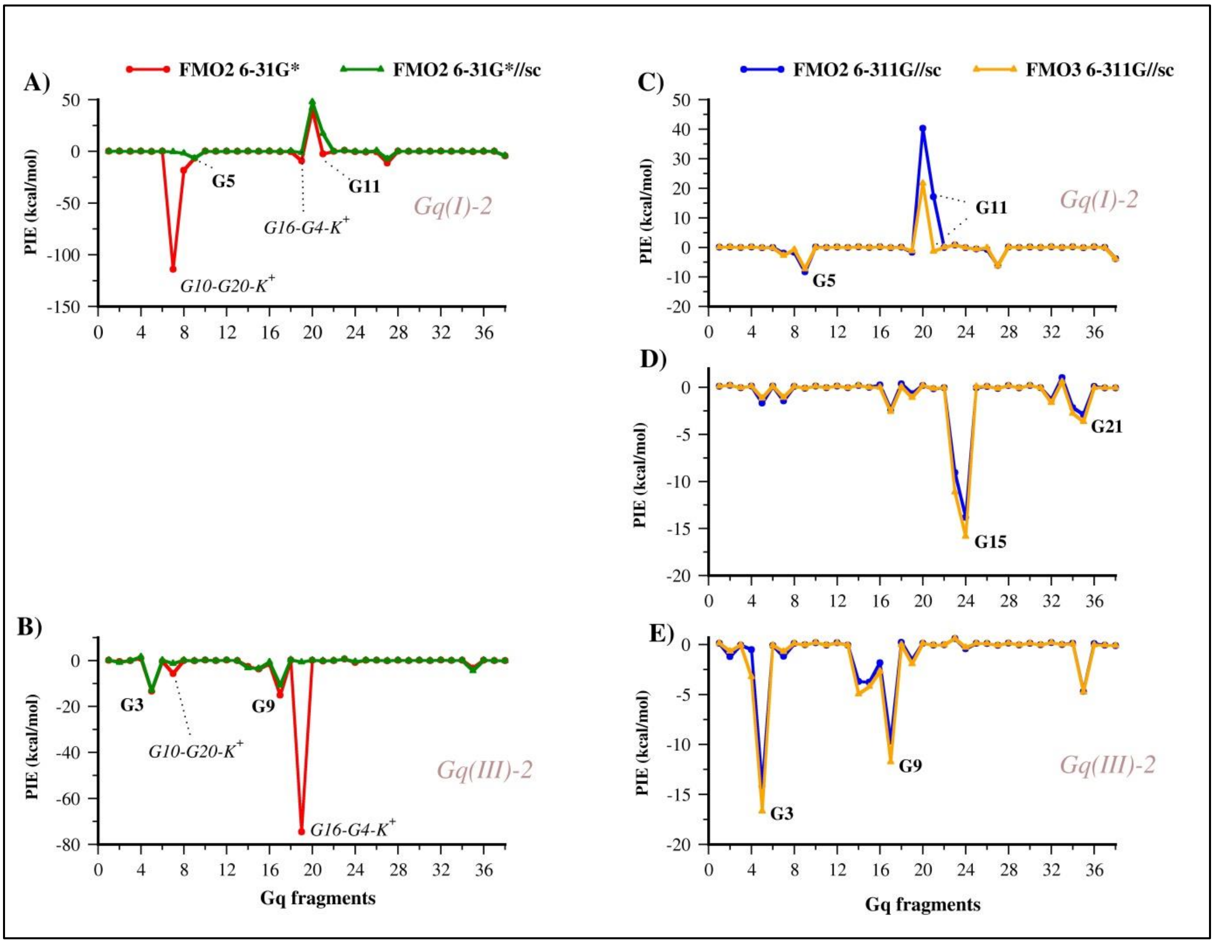

Fig. S6 E_{Li}^{PIE} values for the interaction between Gq fragments and ligand **2** in the binding sites I (A and C), II (D) and III (B and E), computed at the FMO2 RI-MP2/6-31G*, FMO2 RI-MP2/6-31G*//sc, FMO2 RI-MP2/6-311G//sc and FMO3 RI-MP2/6-311G//sc levels of theory and reported by using red, green, blue and lines, respectively

Table S5 EDA of interaction between ligand **1** and the nearest fragment containing K^{+} ion (fragment 7 for Gq(I) and fragment 19 for Gq(II) and Gq(III)) computed at Gq(I) and at FMO2 6-31G*, FMO2 6-31G*//sc, FMO2 6-311G//sc and FMO3 6-311//sc levels of theory. The CT from **1** to fragment 7 or 19, Q^{CT}, is reported in in elementary charge unit. All energy values are in kcal/mol.

**Level of theory**	**PIEDA of *frag7-1* — Gq(I)-1**						
	**PIE**	$\mathbf{E^{es}}$	$\mathbf{E^{ex}}$	$\mathbf{E^{ct}}$	$\mathbf{E^{disp}}$	$\mathbf{E^{sol}}$	$\mathbf{Q^{CT}}$
FMO2 6-31G*	-137.9	-133.186	178.637	-167.483	-10.376	-5.508	-0.2413
FMO2 6-31G*//sc	4.0	42.994	0.049	-0.464	-1.637	-38.405	-0.0138
FMO2 6-311G//sc	2.5	41.759	0.05	-0.588	0.904	-38.147	-0.0183
FMO3 6-311G//sc	2.6	41.759	0.206	-1.079	-0.151	-38.161	-0.0183
	**PIEDA of *frag19-1 — Gq(II)-1***						
FMO2 6-31G*	-76.8	-61.0	91.9	-87.7	-6.2	-13.9	-0.0896
FMO2 6-31G*//sc	0.6	41.0	0.0	-0.3	-0.9	-39.3	-0.011
FMO2 6-311G//sc	1.4	38.5	0.0	-0.4	0.9	-37.6	-0.0145
FMO3 6-311G//sc	0.9	41.0	0.1	-0.5	-0.5	-39.2	-0.0145
	**PIEDA of *frag19-1 — Gq(III)-1***						
FMO2 6-31G*	-78.6	-62.9	89.3	-87.6	-5.9	-11.5	-0.1057
FMO2 6-31G*//sc	0.4	39.9	0.0	-0.4	-0.8	-38.3	-0.0098
FMO2 6-311G//sc	1.3	37.6	0.0	-0.6	0.9	-36.6	-0.0119
FMO3 6-311G//sc	0.7	39.9	0.1	-0.6	-0.4	-38.2	-0.0119

Table S6 EDA of interaction between ligand 1 and the nearest fragment containing K^+ ion computed for the reduced Gq(I)-1 complex (Fig. S4), using the FMO3 (low accuracy) at RI-MP2/6-31G**, RI-MP2/6-311G*, RI-MP2/6-311G**, RI-MP2/6-31G**//sc, RI-MP2/6-311G*//sc and RI-MP2/6-311**//sc levels of theory. The CT from 1 to (G22·G10·K102) fragment, Q^{CT}, is reported in in elementary charge unit. All energy values are in kcal/mol

**Level of theory**	**FMO3 EDA of *(G22·G10· K102)-1 interaction — reduced Gq(I)-1***						
	**PIE**	**E^{es}**	**E^{ex}**	**E^{ct}**	**E^{disp}**	**E^{sol}**	**Q^{CT}**
RI-MP2/6-31G**	-158.0	-122.2	176.9	-193.6	-15.3	-3.7	-0.3696
RI-MP2/6-311G*	-299.9	-181.0	261.1	-284.8	-84.0	-11.2	-0.6598
RI-MP2/6-311G**	-213.6	-132.8	181.4	-246.7	-22.3	6.9	-0.6566
RI-MP2/6-31G**//sc	4.2	46.8	0.1	-0.5	-1.9	-40.3	-0.0044
RI-MP2/6-311G*//sc	5.1	46.7	0.1	-0.33	-0.865	-40.5	-0.0065
RI-MP2/6-311G**//sc	4.2	46.1	0.1	-0.3	-1.5	-40.2	-0.0067

Table S7 EDA of interaction between ligand **2** and the nearest fragment containing K^+ ion (fragment 7 for Gq(I) and fragment 19 for Gq(II) and Gq(III)) computed at Gq(I) and at FMO2 6-31G*, FMO2 6-31G*//sc, FMO2 6-311G//sc and FMO3 6-311//sc levels of theory. The CT from **2** to fragment 7 or 19, Q^{CT}, is reported in in elementary charge unit. All energy values are in kcal/mol

**Level of theory**	**PIEDA *of frag7-1* — Gq(I)-2**						
	**PIE**	E^{es}	E^{ex}	E^{ct}	E^{disp}	E^{sol}	Q^{CT}
FMO2 6-31G*	-114.059	-110.364	164.537	-149.262	-8.262	-10.709	-0.2095
FMO2 6-31G*//sc	-0.424	40.847	0.017	-0.897	-0.787	-39.604	-0.0126
FMO2 6-311G//sc	-1.992	39.224	0.009	-0.935	-1.129	-39.16	-0.0156
FMO3 6-311G//sc	-2.735	39.224	0.123	-1.745	-1.214	-39.123	-0.0156
	**PIEDA of *frag19-1 — Gq(II)-2***						
FMO2 6-31G*	-69.752	-49.403	89.005	-84.81	-5.813	-18.73	-0.140
FMO2 6-31G*//sc	-	-	-	-	-	-	-
FMO2 6-311G//sc	-0.67	39.775	0.006	-0.548	-0.728	-39.175	-0.0161
FMO3 6-311G//sc	-1.081	39.775	0.109	-1.05	-0.785	-39.129	-0.0161
	**PIEDA of *frag19-1 — Gq(III)-2***						
FMO2 6-31G*	-74.525	-54.122	91.088	-89.549	-4.68	-17.262	-0.1219
FMO2 6-31G*//sc	-0.707	40.915	0.007	-0.481	-0.942	-40.206	-0.0104
FMO2 6-311G//sc	-1.55	38.428	0.007	-0.648	-0.636	-38.701	-0.0124
FMO3 6-311G//sc	-1.944	38.428	0.108	-1.228	-0.625	-38.626	-0.0124

Table S8 PIEDA between ligand **1** and underlying nucleobases I, II and III binding sites, computed at the FMO2 RI-MP2/6-31G*, FMO2 RI-MP2/6-31G*//sc, FMO2 RI-MP2/6-311G//sc and FMO3 RI-MP2/6-311G//sc levels of theory. All energy values are reported in kcal/mol

**Binding site: Pair Interaction**	**Level of theory RI-MP2**	**PIEDA**					
		**PIE**	**E^{es}**	**E^{ex}**	**E^{ct}**	**E^{disp}**	**E^{sol}**
Gq(I): 1-DG5	FMO2 6-31G*	-14.3	15.2	19.0	-16.2	-19.4	-12.9
	FMO2 6-31G*//sc	-12.7	1.0	19.8	-6.5	-21.0	-6.0
	FMO2 6-311//sc	-40.5	0.1	24.4	-9.8	-45.5	-9.7
	FMO3 6-311//sc	-32.4	0.1	25.6	-12.0	-36.7	-9.4
Gq(I): 1-DG11	FMO2 6-31G*	-21.6	-16.6	16.5	-8.1	-16.6	3.2
	FMO2 6-31G*//sc	-15.9	-11.8	14.5	-4.9	-16.9	3.1
	FMO2 6-311//sc	-15.9	-11.6	15.6	-5.8	-16.6	2.5
	FMO3 6-311//sc	-16.8	-11.6	15.4	-6.8	-16.4	2.5
Gq(II): 1-DG15	FMO2 6-31G*	-16.2	-6.7	25.4	-11.5	-20.6	-2.6
	FMO2 6-31G*//sc	-13.0	-0.8	20.8	-6.7	-21.4	-4.8
	FMO2 6-311//sc	-13.1	0.2	22.3	-7.8	-21.3	-6.5
	FMO3 6-311//sc	-14.9	0.2	21.8	-9.4	-20.9	-6.6
Gq(II): 1-DG21	FMO2 6-31G*	-23.0	-24.7	10.6	-4.6	-14.3	9.9
	FMO2 6-31G*//sc	-17.3	-20.8	10.6	-4.3	-13.1	10.3
	FMO2 6-311//sc	-16.4	-20.6	11.8	-5.7	-12.3	10.4
	FMO3 6-311//sc	-17.2	-20.6	11.9	-6.9	-12.2	10.6
Gq(III): 1-DG3	FMO2 6-31G*	-15.9	0.1	18.2	-7.8	-21.0	-5.4
	FMO2 6-31G*//sc	-17.0	-0.8	17.4	-6.5	-22.0	-5.0
	FMO2 6-311//sc	-17.5	-0.2	19.1	-8.3	-22.0	-6.1
	FMO3 6-311//sc	-18.9	-0.2	18.9	-9.7	-21.9	-5.9
Gq(III): 1-DG9	FMO2 6-31G*	-18.9	-19.5	12.7	-5.9	-14.8	8.5
	FMO2 6-31G*//sc	-13.0	-15.0	12.2	-4.6	-14.0	8.5
	FMO2 6-311//sc	-12.4	-13.8	13.3	-6.6	-13.6	8.3
	FMO3 6-311//sc	-12.8	-13.8	13.2	-7.3	-13.3	8.3

Table S9 PIEDA between ligand **2** and underlying nucleobases I, II and III binding sites, computed at the FMO2 RI-MP2/6-31G*, FMO2 RI-MP2/6-31G*//sc, FMO2 RI-MP2/6-311G//sc and FMO3 RI-MP2/6-311G//sc levels of theory. All energy values are reported in kcal/mol

**Binding site: Pair Interaction**	**Level of theory RI-MP2**	**PIEDA**					
		**PIE**	**E^{es}**	**E^{ex}**	**E^{ct}**	**E^{disp}**	**E^{sol}**
Gq(I): 2-G5	FMO2 6-31G*	-6.6	-0.6	38.3	-19.1	-19.2	-6.0
	FMO2 6-31G*//sc	-6.7	-9.2	36.7	-9.5	-23.9	-0.7
	FMO2 6-311//sc	-8.3	-10.3	38.9	-10.8	-24.8	-1.3
	FMO3 6-311//sc	-7.3	-10.3	38.0	-11.0	-22.9	-1.1
Gq(I): 2-G11	FMO2 6-31G*	-2.5	-55.4	101.3	-24.5	-27.9	3.9
	FMO2 6-31G*//sc	17.2	-33.0	82.5	-5.5	-29.9	3.0
	FMO2 6-311//sc	17.1	-34.1	85.3	-8.0	-28.8	2.7
	FMO3 6-311//sc	-1.3	-34.1	84.0	-24.8	-28.8	2.4
Gq(II): 2-G15	FMO2 6-31G*	-15.9	-11.5	25.4	-11.6	-19.3	1.1
	FMO2 6-31G*//sc	-	-	-	-	-	-
	FMO2 6-311//sc	-13.8	-6.5	22.3	-8.4	-19.8	-1.4
	FMO3 6-311//sc	-15.8	-6.5	22.0	-10.4	-19.6	-1.3
Gq(II): 2-G21	FMO2 6-31G*	-12.8	-29.2	37.1	-10.1	-17.7	7.1
	FMO2 6-31G*//sc	-	-	-	-	-	-
	FMO2 6-311//sc	-2.9	-23.3	38.4	-10.2	-14.9	7.2
	FMO3 6-311//sc	-3.6	-23.3	38.2	-11.0	-14.9	7.3
Gq(III): 2-G3	FMO2 6-31G*	-13.3	-13.6	43.9	-12.0	-30.7	-0.9
	FMO2 6-31G*//sc	-13.0	-12.8	42.5	-10.5	-31.8	-0.4
	FMO2 6-311//sc	-14.2	-15.8	47.4	-14.0	-30.9	-0.8
	FMO3 6-311//sc	-16.7	-15.8	46.7	-16.1	-30.9	-0.6
Gq(III): 2-G9	FMO2 6-31G*	-15.1	-12.9	3.7	-3.3	-8.9	6.2
	FMO2 6-31G*//sc	-10.7	-8.8	3.5	-4.1	-8.0	6.6
	FMO2 6-311//sc	-9.7	-6.9	3.9	-5.2	-7.6	6.0
	FMO3 6-311//sc	-11.7	-6.9	4.0	-7.0	-8.1	6.2

Table S10 Average values of E^{INT} and of E^{PIE} between ligand **1** and **2** and the underlying nucleobases, computed at FMO2 RI-MP2/6-31G*//sc, FMO2 RI-MP2/6-311G//sc and FMO3 RI-MP2/6-311G//sc levels of theory. All energy values are reported in kcal/mol

**Energy**	**Level of theory: RI-MP2**		
	**FMO2 6-31G*//sc**	**FMO2 6-311G//sc**	**FMO3 6-311G//sc**
		Gq-1 complex	
# average E^{INT}	-34.9	-45.7	-50.2
* Average E^{PIE} (G_x•G_y-1)	-29.6	-38.9	-37.7
		Gq-2 complex	
# average E^{INT}	5.4	-12.9	-30.2
* Average E^{PIE} (G_x•G_y-2)	-6.6	-22.0	-18.8

for each level of theory the average E^{INT} is computed as
[E^{INT}(Gq(I)-ligand)+ E^{INT}(Gq(II)-ligand)+ E^{INT}(Gq(III)-ligand)]/3
* for each level of theory the average E^{PIE} (G_x•G_y-ligand) is computed as
[(E^{PIE}(ligand-G5)+ E^{PIE}(ligand-G11))+ (E^{PIE}(ligand-G15)+ E^{PIE}(ligand-G21))+ (E^{PIE}(ligand-G3)+ E^{PIE}(ligand-G9))]/3

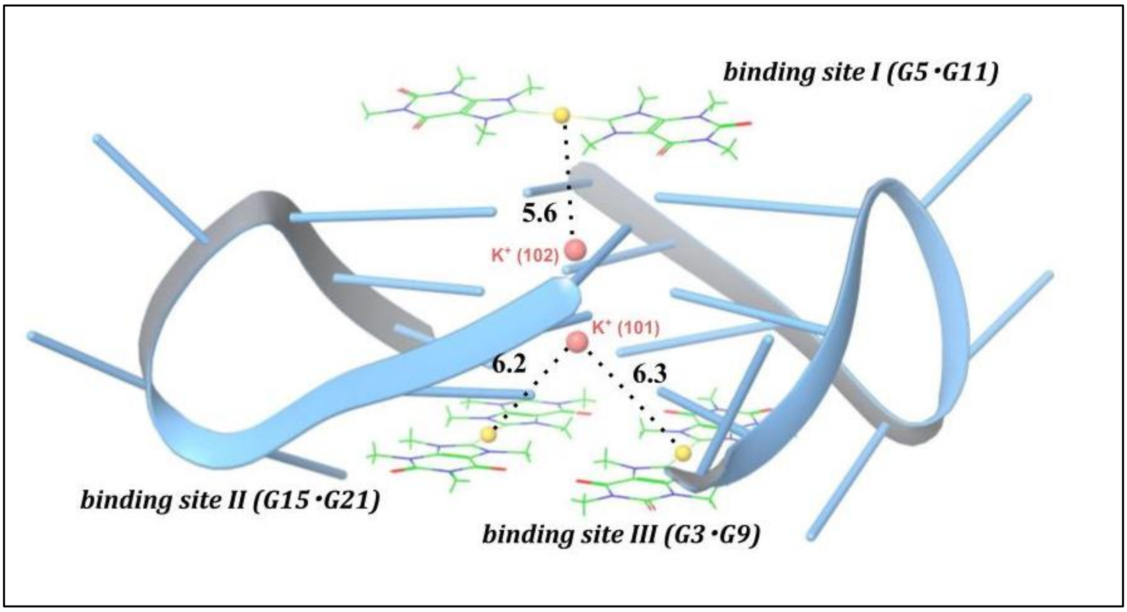

Fig. S7 Distances (dashed lines) between Au(I) and the nearest K^+ ion for ligand 1 in the three binding sites. All values are reported in Å

Table S11 ΔE^{FMO} values computed for Gq-1 and Gq-2 complexes at FMO2 RI-MP2/6-31G*, FMO2 FMO2 RI-MP2/6-31G*//sc and FMO2 RI-MP2/6-311G//sc levels of theory. All values are in kcal/mol

Complex	ΔE^{FMO}		
	**FMO2 RI-MP2/6-31G***	**FMO2 RI-MP2/6-31G*//sc**	**FMO3 RI-MP2/6-311G//sc**
Gq(I)-1	-319.7	2.6	31.0
Gq(II)-1	-84.9	44.5	19.3
Gq(III)-1	-106.5	6.3	-0.3
average	**-170.4**	**17.8**	**16.7**
Gq(I)-2	-175.5	57	16.6
Gq(II)-2	-122.4	- #	-26.8
Gq(III)-2	-138.2	-22.5	-37.7
average	**-145.4**	**17.3**	**-16.0**

ΔE not computed due to unrecoverable issue with SCF convergence of some fragments in LR complexes
